# Quercetin improves retinal glycolysis to slow myopia progression through orchestrating the AKT/FOXO/HK2 axis

**DOI:** 10.1016/j.redox.2026.104139

**Published:** 2026-03-31

**Authors:** Ruixue Zhang, Miao Zhang, Yunxiao Xie, Huixia Wei, Zhaohui Yang, Ying Wen, Jiawen Hao, Yongle Du, Yuanting Yang, Xuewei Yin, Yinqiao Zhang, Wenjun Jiang, Hongsheng Bi, Dadong Guo

**Affiliations:** a Shandong University of Traditional Chinese Medicine, Jinan, 250002, China; b School of Ophthalmology, Shandong First Medical University & Shandong Academy of Medical Science, Jinan, 250000, China; c Affiliated Eye Hospital of Shandong University of Traditional Chinese Medicine, Jinan, 250002, China; d Medical College of Optometry and Ophthalmology, Shandong University of Traditional Chinese Medicine, Jinan, 250002, China; e Shandong Provincial Key Laboratory of Integrated Traditional Chinese and Western Medicine for Prevention and Therapy of Ocular Diseases, Shandong Academy of Eye Disease Prevention and Therapy, Jinan, 250002, China

**Keywords:** Myopia, Glycolysis, Neuron, Single-cell RNA sequencing, Oxidative damage

## Abstract

**Objectives:**

Myopia is a major global public health issue. The retina relies on glycolysis to maintain its normal physiological functions and is vulnerable to oxidative damage. However, the effects of altered glycolysis on myopia progression remain poorly understood. This study aimed to explore how oxidative damage caused by changes in retinal glycolysis promotes myopia and evaluate the potential of quercetin in alleviating this process.

**Methods:**

We used single-cell RNA sequencing to analyze the retinas of myopic guinea pigs, followed by proteomic and phosphoproteomic profiling to identify differentially expressed proteins. We investigated the regulatory role of the AKT/FOXO/HK2 pathway in glycolysis via coimmunoprecipitation and dual-luciferase assays. Additionally, we assessed the role of glycolysis in myopia by overexpressing HK2 and using 2-deoxy-d-glucose (2DG). The effects of neuronal injury on myopia progression were explored using Fos overexpression and knockdown. Finally, after quercetin intervention, we conducted metabolomic analysis and measured retinal mitochondrial pressure and glycolysis rate to evaluate their effects on retinal metabolism.

**Results:**

Multiomics analysis showed that the AKT/FOXO/HK2 pathway suppresses glycolysis during myopia progression. Protein interaction and dual-luciferase assays confirmed that reduced glycolysis promotes oxidative phosphorylation, leading to retinal oxidative damage and accelerating the progression of myopia. Quercetin treatment inhibited the AKT/FOXO/HK2 axis, restored mitochondrial oxygen consumption and glycolysis rates, and mitigated oxidative damage, which subsequently suppressed the activation of stress-responsive Fos. Furthermore, Fos overexpression amplified retinal neuronal injury, apoptosis, and mitochondrial damage to drive myopia progression. Inhibiting Fos expression or quercetin treatment alleviated neuronal injury and apoptosis, thereby inhibiting myopia progression.

**Conclusion:**

Our findings suggest that quercetin may attenuate myopia progression, at least in part, by modulating the AKT/FOXO/HK2 axis to restore retinal glycolysis, thereby reducing oxidative stress and mitigating retinal neuronal damage and apoptosis. This study lays the groundwork for further exploration metabolic reprogramming in myopia pathogenesis.

## Graphical abstract

**Figure:**
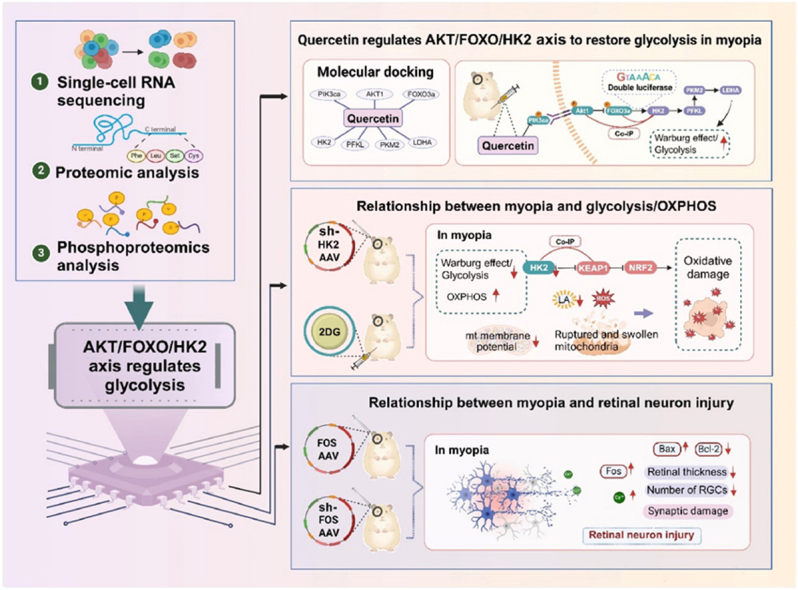

## 1 Introduction

Myopia is one of the most prevalent ocular conditions in children and adolescents worldwide and poses a significant public health challenge. The global prevalence of myopia is rapidly increasing, with projections indicating that by 2050, 49.8% of the population will be affected, and 9.8% will experience high myopia [1]. Despite this growing burden, the underlying mechanisms of myopia remain poorly understood.

The retina plays a critical role in the initial processing of visual information and relies on aerobic glycolysis (the Warburg effect) to convert glucose into lactate, even under oxygen-rich conditions [2]. This metabolic pathway is also predominant in retinal neurons and protects them from oxidative damage [3]. In diabetic retinopathy, altered glycolysis reduces the antioxidant capacity of neurons, contributing to retinal neuronal loss [4]. However, the specific role of glycolysis in myopia progression remains unclear. We hypothesize that changes in aerobic glycolysis within the retina may impair neuronal function, thereby driving the progression of myopia.

Even under aerobic conditions, some cells rely primarily on glycolysis rather than oxidative phosphorylation (OXPHOS), a phenomenon known as aerobic glycolysis or the “Warburg effect.” This metabolic pathway is preferentially employed by both the retina and tumors. As part of the central nervous system, the retina is highly metabolically active and relies on glucose and glycolysis for its survival and function [5]. Approximately 80% of glucose is converted to lactate via glycolysis [6], and lactic acid levels increase by 50% in adult retinas under anaerobic conditions (the Pasteur effect) [7]. A study demonstrated that early mouse eye progenitor cells exhibit increased glycolytic activity and lactic acid production and that the inhibition of glycolysis halts optic nerve vesicle development. However, supplementation with lactic acid restores progenitor cell development [3]. The phosphatidylinositol-3-kinase (PI3K) pathway is activated in myopia [8]. While PI3K/AKT signaling typically promotes metabolism, it is also a canonical inhibitor of FOXO transcription factors [9], which we hypothesize may paradoxically suppress glycolytic enzymes in this context. However, the role of the PI3K/AKT/FOXO pathway in regulating glycolysis and its impact on retinal neurons in myopia remain unclear.

Vision formation begins with the production of visual nerve impulses in the retina, where photoreceptor, bipolar, and ganglion cells transmit information to the visual center along the optic pathway. Neurons are believed to rely primarily on OXPHOS for glucose metabolism. However, research indicates that the transition from aerobic glycolysis to OXPHOS in neuronal cell bodies can lead to oxidative damage and progressive neuronal loss [3]. Thus, while neurons utilize glycolysis to meet energy demands, protective mechanisms are required to mitigate OXPHOS-induced damage [3]. Although neurons primarily utilize aerobic glycolysis for energy, particularly under conditions of high metabolic demand, OXPHOS remains essential for mitochondrial function and synaptic transmission [10]. In diabetic retinopathy, PFKFB3-driven glycolysis impairs the antioxidant capacity of neurons, contributing to retinal neuronal loss [4]. However, it remains unclear whether alterations in glycolysis influence the progression of myopia.

This study demonstrated that in the myopic retina, there is a decrease in glycolysis and an increase in oxidative phosphorylation, as revealed through single-cell sequencing, proteomics, and phosphoproteomics. These findings were further validated by several key points: 1) Quercetin was shown to regulate the PI3K/AKT/FOXO pathway, influencing both glycolysis and oxidative phosphorylation; 2) the effects of OXPHOS and oxidative stress on retinal neurons in myopia were confirmed; and 3) the relationship between neuronal injury and the progression of myopia was investigated using AAV-mediated knockout and overexpression of Fos. This study revealed that quercetin inhibits the AKT/FOXO/HK2 axis, modulates glycolysis and OXPHOS, reduces oxidative stress, and consequently potentially protecting retinal neurons from damage associated with myopia.

## 2 Materials and methods

### 2.1 Ethics and animals

This study received approval from the Experimental Animal Ethics Review of the Affiliated Hospital of Shandong University of Traditional Chinese Medicine (AWE-2022-055) and strictly adhered to the ARVO Statement for the Use of Animals in Ophthalmic and Vision Research.

Healthy post-natal day 14 (P14) guinea pigs (*Cavia porcellus*) were obtained from Jinan Jinfeng Experimental Animal Company. Prior to the study, all animals underwent ophthalmic examinations, and those with pre-existing cataracts or corneal opacities were excluded. This starting age (P14) was specifically chosen to represent the onset of the juvenile period in this precocial species, corresponding to the peak window for visual development and ocular growth. To induce myopia, guinea pigs wore −6.0 D lenses on their right eyes (lens-induced myopia, LIM), while the left eyes served as internal controls. Animals in the normal control (NC) group received no treatment. The induction period lasted for 4 to 6 weeks, a duration strategically selected to observe the progression from early metabolic adaptation to chronic pathological reprogramming and to ensure the development of stable axial elongation and structural remodeling.

To evaluate the effect of quercetin on myopia progression, we used quercetin with a purity of ≥98.06% (HY-18085, MCE, China) to treat LIM animals. Briefly, quercetin was dissolved in 0.1% dimethyl sulfoxide (DMSO) and administered intraperitoneally at low, medium, and high doses of 35 mg/kg (Que-L), 45 mg/kg (Que-M), and 60 mg/kg (Que-H) [[11], [12], [13]], and 0.1% DMSO served as the solvent control (animal number (n) = 159). The administration frequency is once a day throughout the entire myopia induction period (4 weeks or 6 weeks).

To investigate the role of glycolysis in myopia, we administered HK2-carried adeno-associated virus (AAV) (OBiO, China) via intraocular injections at volumes of 1 μL, 3 μL, and 5 μL, corresponding to the HK2-1 μL group, HK2-3 μL group, and HK2-5 μL group, respectively. An empty vector carrying AAV served as the vector control (empty). Additionally, animals in the NC and LIM groups received intraperitoneal injections of 99.93% pure 2-Deoxy-d-glucose (2DG) (HY-13966, MedChemExpress, China) dissolved in PBS at a dose of 500 mg/kg [14,15]; these groups were designated the NC+2DG group and LIM+2DG group, respectively. Moreover, sterilized PBS was used as the control (n = 231). Intraperitoneal injections were administered three times weekly throughout the myopia induction period (4 or 6 weeks). During the experimental period, all animals maintained normal activity levels, food intake, and water consumption. Systematic health monitoring revealed no signs of significant distress or mortality attributable to 2-DG treatment.

To investigate the effect of the Fos gene on retinal neurons in myopia, we performed Fos knockdown using shRNA-Fos (CCTGTCTAGTTCATTCTAT) with a viral titer of 1.14 × 10^12^ vectors per mL (VG/mL) (OBiO, China). Intraocular injections of 1 μL, 3 μL, and 5 μL of sh-Fos-AAV were administered to LIM guinea pigs, which were designated the LIM + shRNA-Fos-carried AAV/1 μL group (sh-Fos-1 μL), the sh-Fos-3 μL group, and the sh-Fos-5 μL group, respectively. Additionally, overexpressed Fos-carrying AAV (5 μL) (OBiO, China) was injected into the intravitreal cavity of NC and LIM animals, which were designated the NC + Fos group and the LIM + Fos group, respectively. An empty vector served as the vector control (n = 147). The dosages for AAV-HK2 and sh-Fos (1 μL, 3 μL, and 5 μL) were determined based on preliminary titration experiments and established protocols for guinea pig intravitreal injections to achieve optimal transduction efficiency while maintaining intraocular pressure stability.

### 2.2 Intravitreal injection

Intravitreal injections were performed under sterile conditions using a surgical microscope. Guinea pigs were anesthetized via isoflurane inhalation, and topical analgesia was achieved by instilling 0.5% proparacaine into the conjunctival sac. Prior to the procedure, pupils were fully dilated with 0.5% compound tropicamide, and the periocular area and conjunctival sac were disinfected with 5% povidone-iodine. With the animal in a lateral recumbent position, a 33-gauge, 15-mm Hamilton microsyringe (Model 7803-05) was inserted perpendicularly through the sclera, 2 mm posterior to the temporal limbus. Upon reaching the vitreous cavity—indicated by a characteristic loss of resistance—the needle was tilted toward the posterior pole. The needle tip was visualized in real-time through the cornea to ensure the avoidance of lenticular or retinal injury. A specified volume (1, 3, or 5 μL) [16,17] of AAV viral suspension or control vehicle was slowly infused, and the needle was held in place for approximately 30 s post-injection to prevent reflux. Following the procedure, ofloxacin ophthalmic ointment was applied to the ocular surface to prevent infection. Animals were placed on a heating pad during recovery. Postoperative ocular examinations were conducted daily; any animals exhibiting significant structural damage, such as cataracts, intraocular hemorrhage, or retinal detachment, were excluded from the study.

### 2.3 Measurement of ocular parameters

#### 2.3.1 Axial length measurement

After 4- and 6-week myopia induction, the axial length of the eyeball was measured using ophthalmic type An ultrasonography (Cinescan, Quantel Medical, France). The instrument parameters were set as follows: the probe frequency was 11 MHz, and the ultrasonic propagation speeds were as follows: the anterior chamber 1557 m/s, the lens 1723 m/s, and the vitreous body 1540 m/s. The final measurement was determined by averaging 10 individual measurements.

#### 2.3.2 Refraction measurement

Following the 4- or 6-week myopia induction period, the negative lenses were meticulously removed, and the guinea pigs were maintained in a dark environment prior to assessment. To ensure maximal mydriasis and cycloplegia, 0.5% compound tropicamide (Sinqi Pharmaceutical, Shenyang, China) was administered topically to the conjunctival sac three times at 5-min intervals. Refractive error was measured 30 to 45 min after the final instillation using a high-precision infrared photorefractor (Striatech, Germany) specifically calibrated for small animal ocular research. To guarantee data accuracy and reproducibility, at least six independent refractive measurements were performed for each eye, with the mean value recorded as the final refractive status.

### 2.4 Single-cell RNA sequencing

#### 2.4.1 Animal tissues and sequencing

Guinea pigs received different treatments for 4 weeks, followed by single-cell RNA sequencing (scRNA-seq) of both the retina and visual cortex and single-nucleus RNA sequencing (snRNA-seq) of the visual cortex (n = 3).

#### 2.4.2 Data processing

After processing with the Cell Ranger pipeline (version 3.1.0) using the guinea pig reference genome Cavpor 3.0, data analysis was performed using the Seurat package (version 4.3.0.1) in R (version 4.3.1). Batch effects among different samples were corrected in the principal component (PC) space using the RunHarmony function of the Harmony package (version 1.0.1) with default parameters. In this study, the top 20 harmony components were further analyzed with the RunUMAP function to embed and visualize the cells in a two-dimensional map.

#### 2.4.3 Data analysis after single-cell RNA sequencing

All the data were statistically analyzed using R (version 4.3.1). The differentially expressed genes (DEGs) in each cluster were identified to annotate the cell clusters. These clusters were then annotated on the basis of DEGs combined with curated known cell markers from the literature, and clusters expressing the same cell markers were merged.

The enriched biological functions of DEGs in each cell type were analyzed using the enriched GO function of the clusterProfiler package (version 4.8.3) in R. Enriched GO terms were filtered with p values adjusted via the Benjamini‒Hochberg (BH) method (adjusted p value < 0.05).

### 2.5 Proteomics

5.1 The lysate (including protease inhibitors and phosphorylase inhibitors) was added to the sample and then mixed by vortexing. The lysate was shaken and ground three times with a high-throughput tissue grinder and subsequently lysed on ice for 30 min, followed by centrifugation at 4 °C and 12000×*g* for 20 min. Finally, the supernatant was collected, and BCA quantification was performed.

5.2 Two microliters of 0.5 M TCEP was added to 1 mg of protein, and the mixture was maintained at 37 °C for 1 h. Then, 4 μL of 1 M iodoacetamide was added, and the mixture was incubated at room temperature for 40 min in the dark. Next, precooled acetone was added at a volume-to-sample ratio of 1:5, and the sample was precipitated at −20 °C overnight. The supernatant was then discarded by centrifugation at 12000×*g* at 4 °C for 20 min. Next, 1 mL of 90% precooled acetone solution was added to the sample, which was then cleaned by vortex mixing. Afterward, the supernatant was discarded following centrifugation at high speed (12000×*g* at 4 °C for 20 min). This cleaning procedure was repeated twice. Once it was confirmed that the acetone had completely evaporated from the precipitate, the residue was redissolved in 100 μL of 100 mM TEAB. Then, trypsin (Promega, Madison, WI) was added at a mass ratio of 1:50, and enzymatic hydrolysis was performed at 37 °C overnight. Finally, the sample was desalted using a C18 column, and the final peptide concentration was determined with a peptide profiling kit (Pierce™ 23275). The sample was then freeze-dried.

5.3 The lyophilized peptides were dissolved in 0.1% formic acid solution and analyzed by LC‒MS/MS. The complete system was a timsTOF Pro 2 mass spectrometer (Bruker Daltonics) in series with an UltiMate 3000 system (Thermo Fisher Scientific, MA, USA). A total of 200 ng samples were taken (analysis column: 25 cm × 75 μm i.d., IonOpticks), the samples were separated on a 60 min gradient, and the column temperature was 50 °C. The column flow rate was set to 500 nL/min, and the gradient started at the 4% B phase, increased to 28% in 45 min, 44% in 4 min, and 90% in 4 min, was maintained for 3 min, and 4% equilibrium was reached for 4 min.

5.4 We set the diaPASEF mode for DIA data acquisition, the mass spectrometer scanned from 349 to 1229 *m*/*z*, and the isolation window width was set to 40 Da. During PASEF MS/MS scanning, the impact energy increases linearly with ion mobility. From 59 eV (1/K0 = 1.6 Vs/cm2) to 20 eV (1/K0 = 0.6 Vs/cm2), the DIA data were analyzed using the Spectronaut 19 default parameter (BGS Factory Settings (default)). The sequence database used was UniProt-Cavia_porcellus (version 2022, 18248 entries) with trypsin hydrolysis (n = 3).

### 2.6 Phosphoproteomics

Phosphoproteomic analysis was conducted following the first three steps of the general proteomics workflow. Briefly, lyophilized peptides were processed using a HiSelectTM TiO2 Phosphopeptide Enrichment Kit (Thermo Fisher Scientific, MA, USA) to enrich phosphorylated peptides according to the manufacturer's instructions. After enrichment, the samples were immediately vacuum dried. All subsequent steps were consistent with those employed in the general proteomics protocol (n = 3).

### 2.7 Molecular docking and protein interactions

In this study, potential target interactions were predicted using the STITCH database. The STITCH database provides protein‒protein and protein‒compound interaction data on the basis of experimental evidence, predictive algorithms, and literature support, with each interaction assigned a confidence score. To confirm the target interactions, the following criteria were applied. First, interactions with higher confidence scores were prioritized for analysis, as a higher score indicates greater reliability. Second, interactions reported in the literature were given preference to ensure the credibility of the data. Third, the predicted interactions from the STITCH database were combined, and targets that yielded consistent results across the computational models were selected. Finally, cross-validation was performed to ensure the consistency of the candidate targets across multiple databases. These methods allowed us to confirm the potential interactions of PIK3Ca, AKT1, FOXO3a, HK2, PFKL, PKM2, and LDHA, providing a reliable basis for subsequent molecular docking analysis. The 3D structures of PIK3Ca, AKT1, FOXO3a, HK2, PFKL, PKM2, and LDHA were retrieved from the PDB database and preprocessed via PyMOL software. The docking grid box was configured with AutoDockTools software to encompass the active site regions of the proteins. Molecular docking was performed using AutoDock Vina, and postdocking analysis was conducted using PyMOL software.

### 2.8 Dual-luciferase

Bioinformatics analysis identified complementary binding sites between FOXO3a and the HK2 gene. Subsequently, wild-type (WT) and mutant (MT) dual-luciferase vectors containing the HK2 gene 3′UTR were constructed. The cells were transfected with either FOXO3a mimics or negative control mimics followed by measurement of the relative luciferase activity (n = 3).

### 2.9 Preparation of primary retinal single-cell suspensions

To obtain retinal cells for Seahorse metabolic flux analysis, ROS detection, and mitochondrial membrane potential assays, an optimized enzymatic dissociation protocol was employed. Briefly, guinea pig eyeballs were enucleated under sterile conditions, and the retinal tissues were rapidly isolated. After rinsing in ice-cold phosphate-buffered saline (PBS), the retinas were minced into approximately 1 mm^3^ fragments. The tissue was then digested with 0.25% Trypsin-EDTA (Gibco, USA) at 37 °C for 20 min, with gentle pipetting every 5 min to facilitate dissociation. Proteolysis was terminated by adding an equal volume of DMEM/F12 medium supplemented with 10% fetal bovine serum (FBS).

The resulting cell suspension was filtered through a 70-μm cell strainer (BD Biosciences) to remove undissociated tissue aggregates and centrifuged at 300×*g* for 5 min at 4 °C. After discarding the supernatant, the cell pellet was resuspended according to downstream experimental requirements: in Seahorse XF Base Medium for metabolic assays, or in PBS for flow cytometry and fluorescence detection. This suspension represents a heterogeneous retinal cell population—comprising photoreceptors, bipolar cells, Müller glia, and ganglion cells—that maintains its endogenous cellular composition.

### 2.10 ROS detection

Reactive oxygen species (ROS) levels were quantified using a commercial ROS Assay Kit (S0033 M, Beyotime, China) according to the manufacturer's instructions. Briefly, primary retinal single-cell suspensions were prepared as described in Section 9 of this chapter. The cells were resuspended in PBS and incubated with the DCFH-DA probe at a final concentration of 10 μM. Incubation was performed at 37 °C for 20 min in a cell culture incubator, protected from light. To ensure optimal probe-cell contact, the tubes were gently inverted every 5 min. Following incubation, the cells were washed three times with PBS to thoroughly remove extracellular probes. ROS-associated fluorescence was immediately detected via flow cytometry (n = 4).

### 2.11 Mitochondrial membrane potential detection

The mitochondrial membrane potential was detected using the JC-1 mitochondrial membrane potential detection kit (C1071 M, Beyotime, China). The brief steps are as follows: Prepare the primary retinal single-cell suspension according to the method described in Section 9 of this chapter. Prepare the JC-1 staining working solution according to the instructions of the kit. Mix the cell suspension with the JC-1 working solution thoroughly and incubate it in a 37 °C cell culture box under light for 20 min. After incubation, wash the cells twice with JC-1 staining buffer (1 × ). Then, immediately perform the detection using a flow cytometer.

### 2.12 LA detection

The tissue samples were accurately weighed and homogenized with nine times their volume of normal saline (weight [g]: volume [mL] = 1:9). The homogenate was centrifuged, and the supernatant was collected. The absolute OD was measured in the 10% homogenate supernatant, and the LA activity was assessed according to the instructions of the LA kit (A019-2-1, NJJCbio, China) (n = 6).

### 2.13 LDHA detection

The tissue samples were homogenized in an ice bath at a ratio of 1 g of tissue to 5 mL of extraction buffer. The homogenate was then centrifuged, and the supernatant was collected. Finally, lactate dehydrogenase (L-LDH) activity was measured according to the instructions provided with the L-LDH kit (BC0680, Solarbio, China) (n = 6).

### 2.14 Metabolomics

Ultrahigh-performance liquid chromatography‒mass spectrometry (UHPLC‒MS) was performed using an Agilent 1290 Infinity LC system (Agilent, California, USA). For sample preparation, 1 mL of extraction solution (methanol:water, 80:20, v/v) was added to each sample, which was subsequently vortexed for 60 s and subjected to two rounds of low-temperature ultrasonication at 4 °C for 60 min. After incubation at −20 °C for 1 h to precipitate proteins, the samples were centrifuged at 12,000×*g* for 10 min at 4 °C, followed by filtration with a 0.22 μm membrane. Finally, the filtrate was stored at −80 °C until analysis.

UHPLC‒MS analysis was conducted on an Agilent 1290 Infinity LC system coupled with a 6545 Q-TOF mass spectrometer. Chromatographic separation was achieved using a ZORBAX Eclipse Plus C18 column (2.1 mm × 100 mm, 1.8 μm) with a mobile phase consisting of (A) water + 0.1% formic acid and (B) acetonitrile + 0.1% formic acid. The gradient elution conditions were as follows: 0–2 min, 5% B; 2–10 min, 5–95% B; 10–12 min, 95% B; 12–14 min, 95–5% B; and 14–16 min, 5% B, with a flow rate of 0.3 mL/min. MS data were acquired in positive and negative ion modes with an electrospray ionization (ESI) source.

The samples were analyzed in triplicate (n = 6), with quality control samples included for consistency.

### 2.15 Coimmunoprecipitation (co-IP)

Retinal tissue was ground at a low temperature, and the resulting supernatant was obtained by centrifugation. Immunoprecipitation (IP) antibodies were used to bind proteins according to the manufacturer's instructions. Subsequently, 1 mg of protein was added to the beads linked to the IP antibody IgG. The samples were incubated overnight at 4 °C. After the bead‒antibody‒antigen complex formed, the beads were washed with lysis buffer, followed by centrifugation. The final pellet was resuspended in electrophoresis buffer, heated at 95 °C for 15 min, and subjected to SDS‒PAGE followed by immunoblotting (n = 3).

### 2.16 Ca^2+^ flux measurements

Noninvasive microtest technology (NMT) (Younger USA LLC., Amherst, MA, USA) was employed to measure Ca^2+^ fluxes in retinal tissues. Before the measurements, a calcium ion-selective microelectrode was prepared as previously described [18]. The retinal tissues were placed in a clean ionic solution environment within 35 mm culture dishes (Nest, Wuxi, China). After recording for 5 min, the raw data microvolt differences (△μV) were imported and converted into Ca2+ fluxes using JCal V3.3 (n = 3).

### 2.17 Transmission electron microscopy

The left visual cortex of the guinea pig brain was isolated and cut into 1 mm × 1 mm × 1 mm sections. The samples were rinsed, dehydrated, soaked, and embedded in Epon 812. They were then sectioned into 70–100-nm ultrathin slices and stained with lead citrate and uranyl acetate. Finally, the sections were analyzed using a transmission electron microscopy (JEOL-1200 E, Japan) (n = 3). For TEM quantification, we took 10 random microphotographs of each sample. To ensure reproducibility, ultrastructural quantification was performed by two independent observers blinded to the experimental groups using ImageJ software (NIH, Bethesda, MD, USA). Specifically, mitochondria were deemed damaged if they displayed clear signs of matrix swelling (electron-lucency), extensive disorganization or loss of cristae, or structural rupture of the outer/inner membranes. Synaptic length was defined operationally as the contour length of the electron-dense postsynaptic density (PSD). It was measured by manually tracing the continuous electron-dense region of the synapse with the freehand line tool in ImageJ, providing a standardized metric for structural synaptic integrity.

### 2.18 Western blot

For Western blot analysis, the target proteins were separated via 10% SDS‒PAGE (Shandong Sparkjade Biotech., Co., Ltd., Jinan, China) and quantified using the enhanced chemiluminescence method (Sparkjade ECL plus, Jinan, China). Primary antibodies were used to analyze the expression of target proteins (Table 1). Finally, images were acquired using the FUSION-FX7 imaging system (Vilber Lourmat, Marne-la-Vallée, France) and quantified with Fusion CAPT software (Vilber Lourmat, France) (n = 6).

**Table 1:** List of primary antibodies.

Primary antibody	dilution	Lot number & Manufacturer
PIK3Ca	1:1000	bs2067R, Bioss, China
*p*-PIK3Ca	1:1000	bs-5570R, Bioss, China
AKT1	1:1000	bs0115R, Bioss, China
p- AKT1	1:1000	bs-5194R, Bioss, China
FOXO3a	1:5000	10849-1-AP, Proteintech, China
*p*-FOXO3a	1:10000	28755-1-AP, Proteintech, China
HK2	1:20000	22029-1-AP, Proteintech, China
PFKL	1:1000	A7708, ABclonal, China
PKM2	1:1000	A22408, ABclonal, China
LDHA	1:1000	A0861, ABclonal, China
Fos	1:2000	bs-23042R, Bioss, China
Bax	1:20000	50599-2-Ig, Proteintech, China
Bcl-2	1:30000	68103-1-Ig, Proteintech, China
β-actin	1:5000	bs-10966R, Bioss, China

### 2.19 Immunofluorescence staining

The slides were washed with PBS, blocked with BSA and then incubated with primary antibodies (Table 2) overnight at 4 °C. HRP-labeled secondary antibodies (dilution 1:2000, Servicebio, Wuhan, China) were subsequently applied for 50 min. The slides were then incubated with a DAPI dye solution. Finally, the slides were observed under a fluorescence microscope (Nikon Eclipse 55i, Japan) (n = 3).

**Table 2:** Immunofluorescence staining Primary antibody.

Primary antibody	dilution	Lot number & Manufacturer
PIK3Ca	1:300	bs2067R, Bioss, China
AKT1	1:300	bs0115R, Bioss, China
FOXO3a	1:500	10849-1-AP, Proteintech, China
HK2	1:500	22029-1-AP, Proteintech, China
PFKL	1:300	A7708, ABclonal, China
PKM2	1:300	A22408, ABclonal, China
LDHA	1:300	A0861, ABclonal, China
Fos	1:300	bs-23042R, Bioss, China
NRF2	1:500	16396-1-AP, Proteintech, China
KEAP1	1:500	10503-2-AP, Proteintech, China

### 2.20 Hematoxylin–eosin and nissl staining

For hematoxylin‒eosin (H&E) staining, paraffin-embedded sections were first dewaxed and then stained with H&E dye solution, followed by dehydration and subsequent sealing. The stained sections were then examined under a microscope. For Nissl staining, the sections were immersed in the dye solution for 2–5 min, rinsed with water, differentiated using acetic acid and sealed with transparent xylene before microscopic observation. For the quantification of retinal ganglion cells (RGCs) and Fos-positive cells, three non-overlapping fields (200 × 200 μm^2^) per retina section were randomly selected. The number of Nissl-stained neurons and immunofluorescence-positive cells was counted independently by two blinded observers using ImageJ software. Data were expressed as cell density (cells/mm^2^) to ensure statistical robustness across groups (n = 3).

### 2.21 Measurement of cell mitochondrial stress and glycolysis rates

The mitochondrial oxygen consumption rate (OCR) and extracellular acidification rate (ECAR) of primary retinal cells were measured in real time using the Agilent Seahorse XFe 96 extracellular flux analyzer (Agilent Technologies, CA, USA).

#### 2.21.1 Cell seeding and equilibration

The primary retinal cells were resuspended in pre-warmed Seahorse XF DMEM Base Medium (without sodium bicarbonate, pH 7.4), which had been supplemented with 10 mM glucose, 1 mM pyruvate sodium, and 2 mM l-glutamine. The cells were seeded at a density of 20,000 cells per well in an XF96 cell culture microplate coated with Poly-l-Lysine, with a volume of 80 μL per well. To ensure uniform cell adhesion, the microplate was first centrifuged at 200×*g* for 1 min, and then placed in a 37 °C CO_2_-free incubator for 45 min to equilibrate.

#### 2.21.2 Cell mito stress test

This experiment is designed to assess the mitochondrial respiratory function. The following drugs were successively injected into the system: (1) Oligomycin (1.5 μM), an ATP synthase inhibitor, used to measure ATP-coupled respiration; (2) FCCP (1.0 μM), a mitochondrial uncoupling agent, used to measure the maximum respiratory capacity; (3) Rotenone/Antimycin A mixed inhibitor (0.5 μM), a complex I and III inhibitor, used to completely inhibit mitochondrial respiration in order to determine non-mitochondrial oxygen consumption.

#### 2.21.3 Glycolytic rate assay

Sequentially inject the following drugs into the system: (1) Rotenone/Antimycin A mixed inhibitor (0.5 μM), which is used to block the mitochondrial respiratory chain, thereby eliminating the contribution of CO_2_ from mitochondria to the extracellular acidification, forcing the cells to rely on glycolysis for energy supply; (2) 2-Deoxy-d-glucose (2-DG, 50 mM), a hexokinase competitive inhibitor, used to completely inhibit the glycolysis process, thereby establishing the baseline of non-glycolytic acidification.

#### 2.21.4 Normalization

To correct for minor variations in cell number across wells, the medium was aspirated immediately following the assay. Cells were lysed using RIPA buffer, and total protein content per well was quantified using a BCA Protein Assay Kit (Pierce). All oxygen consumption rate (OCR, pmol/min) and extracellular acidification rate (ECAR, mpH/min) values were normalized to the total protein mass (μg protein/well). Data analysis was performed using Seahorse Wave software (version 2.6).

### 2.22 Statistical analysis

A significant difference was determined via GraphPad Prism (v.10.1.2) for experimental data and R (v.4.3.1) with RStudio (v.3.5.3) for additional analyses. Normality was assessed using the Shapiro-Wilk test, while homogeneity of variance was evaluated by the Brown-Forsythe test. To compare two groups, an unpaired two-tailed Student's t-test was employed. When comparing three or more groups, one-way analysis of variance (ANOVA) was performed, followed by Tukey's post-hoc test for multiple comparisons when comparing all groups against each other. Alternatively, Dunnett's post-hoc test was used when comparing several treatment groups to a single control group. Conservations between cell types were evaluated by Pearson correlation analysis on the basis of the mean UMI of expressed genes in each cell cluster, whereas functional enrichment analysis of differentially expressed genes was carried out using the Database for Annotation, Visualization, and Integrated Discovery (DAVID). For differential expression analysis in Seurat, an unpaired two-tailed Student's *t*-test was performed to compare data across the four groups. A p value of <0.05 was considered to indicate statistical significance. Unless otherwise specified, each experiment was repeated three or more times with biologically independent samples (n = 3).

## 3 Results

### 3.1 The PI3K/AKT/FOXO signaling pathway is activated, and glycolysis is decreased in myopia

**Fig. 1:**
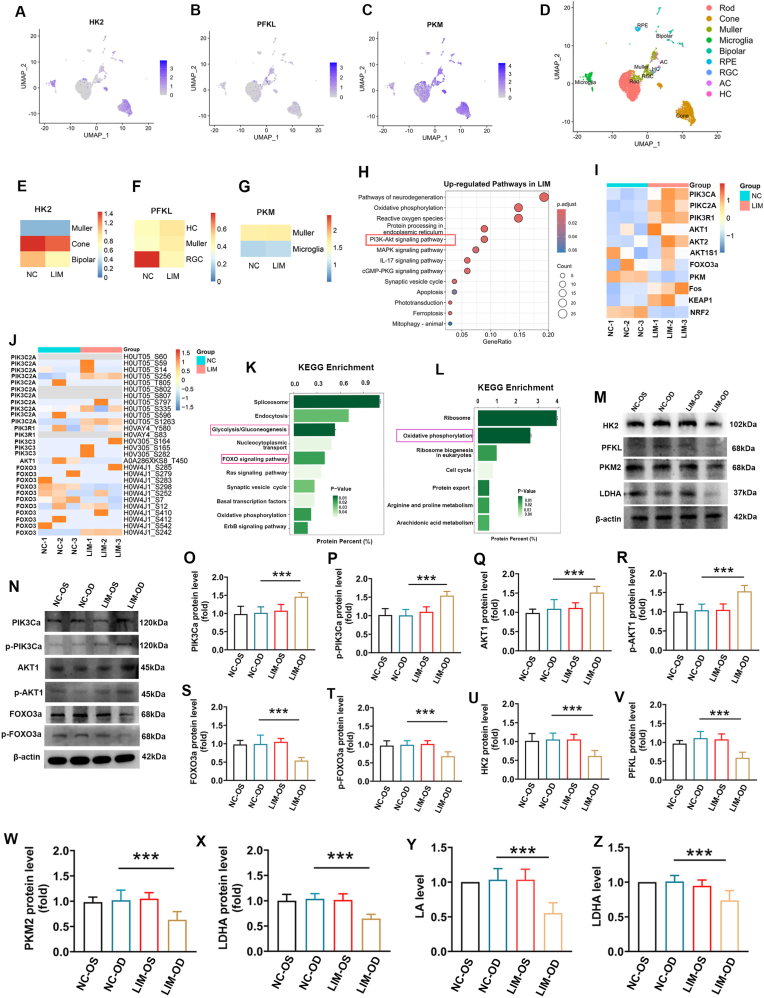
Multi-omics analysis of the regulation of glycolysis by PI3K/AKT/FOXO signaling pathway in myopic retina. A-C. The distribution of HK2, PFKL, and PKM in various retinal cell types was analyzed by single-cell sequencing. D. UMAP visualization of the transcriptomic diversity of the retina. Cell types are shown in different colors. E-G. Heat maps of HK2, PFKL, and PKM expression levels in various retinal cell types were analyzed by single-cell sequencing. H. KEGG analysis of differentially expressed genes in retinal single-cell RNA sequencing. I. Heat map of proteomic analysis results. J. Heat maps of phosphorylated proteomics analysis. K. Phosphorylated proteomics KEGG analysis results. L. Proteomic KEGG analysis results. M. HK2, PFKL, and PKM2 expression were detected by Western blot in NC-OS, NC-OD, LIM-OS, and LIM-OD. Samples derived from the same experiment and that blots were processed in parallel. N. PIK3Ca, *p*-PIK3Ca, AKT1, *p*-AKT1,FOXO3a, and *p*-FOXO3a expression detected by Western blot in NC-OS, NC-OD, LIM-OS, and LIM-OD. Samples derived from the same experiment and that blots were processed in parallel. O-T. Bar graphs of Western blot analysis for PIK3Ca、p- PIK3Ca 、AKT1、p-AKT1, FOXO3a, and *p*-FOXO3a in NC-OS, NC-OD, LIM-OS and LIM-OD (∗∗∗P < 0.001). U-X. Bar graphs of Western blot analysis for HK2, PFKL, and PKM2 in NC-OS, NC-OD, LIM-OS, and LIM-OD (∗∗∗P < 0.001). Y. Bar graphs of LA analysis in NC-OS, NC-OD, LIM-OS and LIM-OD groups (∗∗∗P < 0.001, ∗P < 0.05). Z. Bar graphs of LAHA analysis in NC-OS, NC-OD, LIM-OS and LIM-OD groups (∗∗∗P < 0.001, ∗P < 0.05).

First, single-cell sequencing revealed a decrease in the expression of HK2 (in cone, Muller, and bipolar) (Fig. 1A–E), PFKL (in HC, Muller, and RGC) (Fig. 1B–F), and PKM (Muller, and microglia) (Fig. 1C–G, D) in myopic retinas (Fig. S1, Table S1) (P < 0.05, |log2FC| ≥ 0.25). KEGG analysis indicates that the PI3K/AKT signaling pathway is associated with myopia (Fig. 1H).

Proteomic analysis revealed that a total of 8153 proteins were identified, among which the total number of differentially expressed proteins was 538 (FDR <0.05, |log2FC| ≥ 0.58), including 184 up-regulated proteins and 354 down-regulated proteins. Among the differentially expressed proteins we obtained, 79 are related to the PI3K/AKT/FOXO/HK2 pathway, indicating that the occurrence of myopia leads to the dysregulation of this pathway. Proteomic validation also confirmed elevated PIK3Ca and AKT1 and reduced FOXO3a expression in myopic retinas (Fig. 1I), The results of phosphoproteomic analysis revealed that, in the comparison between the LIM and NC groups, a total of 5335 differentially expressed phosphorylated peptides were detected (FDR <0.05, |log2FC| ≥ 0.58), among which 1622 were upregulated and 3713 were downregulated, phosphoproteomic analysis revealed significantly decreased *p*-FOXO3a levels in myopic retinas (Fig. 1J), KEGG analysis linked the FOXO signaling and glycolysis/gluconeogenesis pathways (phosphoproteomic analysis) to myopia (Fig. 1K), and the oxidative phosphorylation signaling pathway (Proteomic analysis) was associated with myopia (Fig. 1L). The KEGG website predicts that the PI3K/AKT/FOXO signaling pathway regulates glycolysis (Fig. S2). Therefore, we hypothesize that the AKT/FOXO/HK2 pathway may regulate glycolysis in retinal neurons and could play a role in the progression of myopia.

Additionally, Western blot (WB) analysis revealed increased expression of PIK3Ca and AKT1 and decreased expression of FOXO3a in LIM-OD retinas compared with LIM-OS, NC-OD, and NC-OS retinas (all ∗∗∗P < 0.001) (Fig. 1N and O‒T). Additionally, WB results revealed that the levels of the glycolytic enzymes HK2, PFKL, PKM, and LDHA were lower in LIM-OD retinas than in LIM-OS, NC-OD, and NC-OS retinas (all ∗∗∗P < 0.001) (Fig. 1M, U-X). Furthermore, retinal lactate and lactate dehydrogenase levels were significantly reduced in LIM-OD (Fig. 1Y and Z).

### 3.2 The activated PI3K/AKT/FOXO signaling pathway inhibits glycolysis in the retina of myopic Guinea pigs

**Fig. 2:**
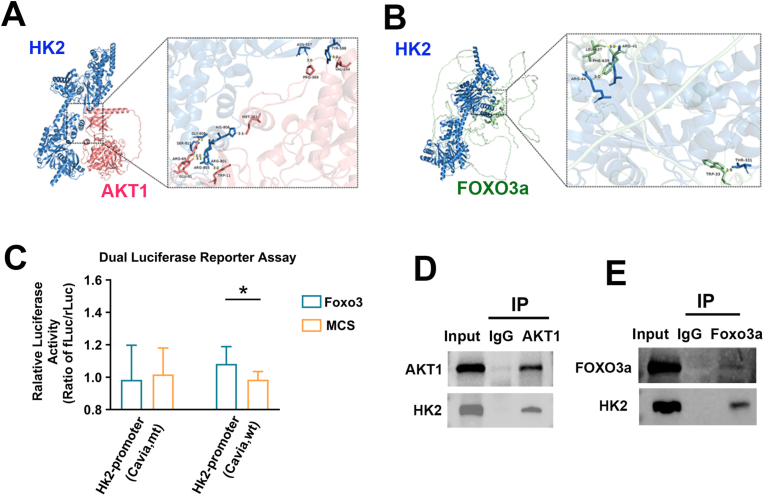
Gene and protein interaction analysis. A, B. AKT1-HK2, FOXO3a-HK2 molecular docking. C. Dual luciferase analysis of FOXO3 regulation of HK2. D. The protein interactions between AKT1 and HK2, FOXO3a and HK2 were analyzed by co-immunoprecipitation.

To determine the effect of the PI3K signaling pathway on glycolysis, we conducted protein interaction predictions for FOXO3a-HK2 and AKT1-HK2. The results revealed significant interactions, with docking scores of −224.06 for HK2-AKT1 and -294.47 for HK2-FOXO3A (Fig. 2A and B).

Next, by leveraging bioinformatics-predicted interaction targets of the FOXO3a and HK2 genes (Table S2), we performed dual luciferase assays to verify the regulatory role of FOXO3a on HK2. Compared with the negative control group, FOXO3a significantly increased the wild-type luciferase activity of the HK2 gene in guinea pig retinas. These results confirm that FOXO3a can effectively modulate the expression of the 3′UTR of the HK2 gene, identifying HK2 as a target gene regulated by FOXO3a (Fig. 2C). Additionally, Co-IP assays demonstrated protein interactions between FOXO3a and HK2, as well as between AKT1 and HK2, in the guinea pig retina (Fig. 2D and E).

### 3.3 Quercetin regulates the AKT/FOXO/HK2 pathway and influences glycolysis to slow myopia progression

**Fig. 3:**
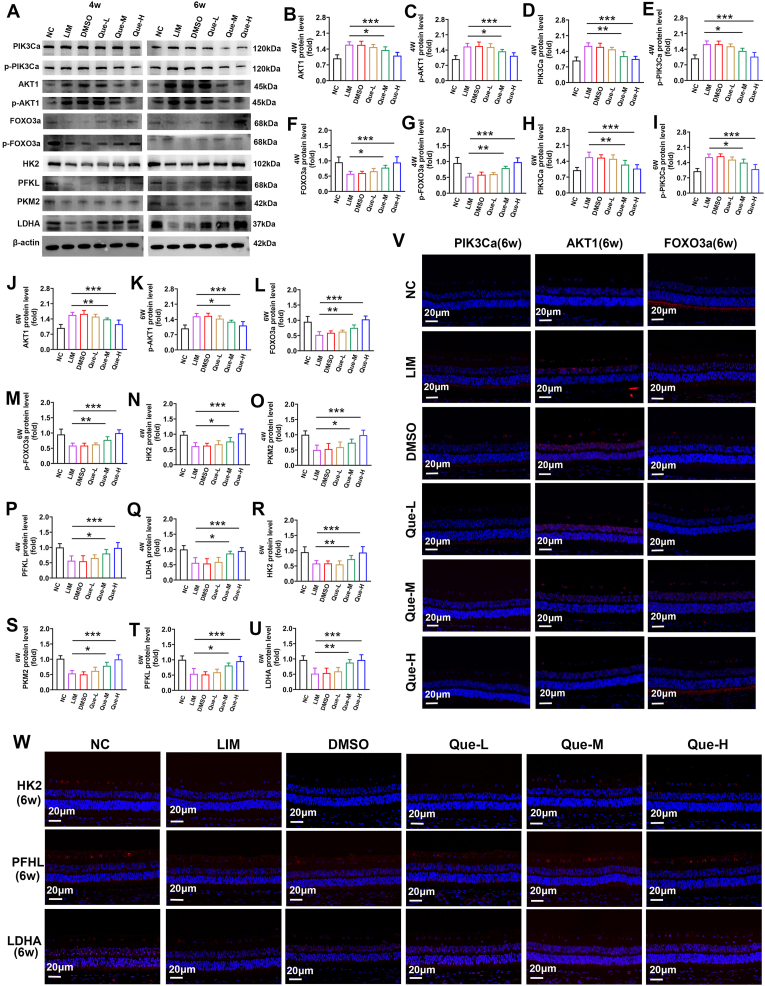
Quercetin inhibits the PI3K signaling pathway and affects glycolysis. A.PIK3Ca、p- PIK3Ca 、AKT1、p-AKT1, FOXO3a, *p*-FOXO3a, HK2, PFKL, PKM2 and LDHA expression detected by Western blot in NC, LIM, DMSO, Que-L, Que-M, Que-H group in 4w and 6w. Samples derived from the same experiment and that blots were processed in parallel. B–U. Bar graphs of Western blot analysis for PIK3Ca、p- PIK3Ca 、AKT1、p-AKT1, FOXO3a, *p*-FOXO3a, HK2, PFKL, PKM2, and LDHA in NC, LIM, DMSO, Que-L, Que-M, Que-H group in 4w and 6w. (∗∗∗P < 0.001). V. PIK3Ca, AKT1, FOXO3a immunofluorescence staining in NC, LIM, DMSO, Que-L, Que-M, Que-H group in 4w and 6w. W. HK2, PFKL, and LDHA immunofluorescence staining in NC, LIM, DMSO, Que-L, Que-M, Que-H group in 4w and 6w.

To investigate the regulation of the AKT/FOXO/HK2 pathway by quercetin, we administered intraperitoneal injections of quercetin to LIM guinea pigs. Molecular docking predictions suggested that quercetin could modulate the AKT/FOXO/HK2 pathway and glycolysis (Fig. S3). Western blot (WB) and immunofluorescence analyses revealed that, compared with the LIM group, quercetin treatment at medium (45 mg/kg) and high doses (60 mg/kg) resulted in decreased expression of PIK3Ca, *p*-PIK3Ca, AKT1, and *p*-AKT1 (all ∗P < 0.05 or ∗∗∗P < 0.001) (Fig. 3A–E, H-K, V), along with increased expression of FOXO3a and *p*-FOXO3a (all ∗∗P < 0.01 or ∗∗∗P < 0.001) (Fig. 3 A, F, G, L, M, V). However, no significant changes were observed in the low-dose group (35 mg/kg), indicating that quercetin inhibits the AKT/FOXO/HK2 axis.

**Fig. 4:**
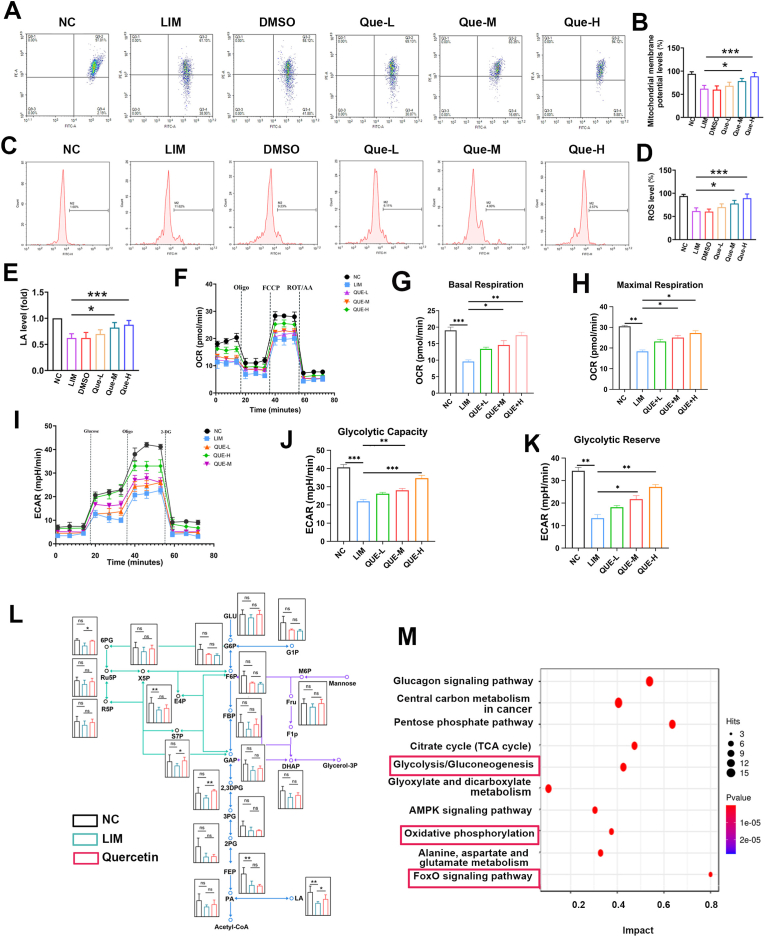
Effect of quercetin on retinal metabolism in myopia。 A. Mitochondrial membrane potential was detected in NC, LIM, DMSO, Que-L, Que-M, and Que-H groups at 6 weeks. B. Bar graphs of Mitochondrial membrane potential analysis in NC, LIM, DMSO, Que-L, Que-M, Que-H group (∗∗∗P < 0.001, ∗P < 0.05). C. ROS were detected in NC, LIM, DMSO, Que-L, Que-M, and Que-H groups at 6 weeks. D. Bar graphs of ROS analysis in NC, LIM, DMSO, Que-L, Que-M, Que-H group (∗∗∗P < 0.001, ∗P < 0.05). E. Bar graphs of LA analysis in NC, LIM, DMSO, Que-L, Que-M, Que-H group (∗∗∗P < 0.001, ∗P < 0.05). F. Mitochondrial respiratory function in the retina after 6 weeks of myopia induction. G. Bar graphs of basal mitochondrial respiration analysis in NC, LIM, Que-L, Que-M, Que-H group (∗∗∗P < 0.001, ∗∗P < 0.01, ∗P < 0.05). H. Bar graphs of maximal mitochondrial respiration analysis in NC, LIM, Que-L, Que-M, Que-H group (∗∗∗P < 0.001, ∗∗P < 0.01, ∗P < 0.05). I. Glycolytic function in the retina after 6 weeks of myopia induction. J. Bar graphs of glycolytic capacity analysis in NC, LIM, Que-L, Que-M, Que-H group (∗∗∗P < 0.001, ∗∗P < 0.01). K. Bar graphs of glycolytic reserve analysis in NC, LIM, Que-L, Que-M, Que-H group (∗∗P < 0.01, ∗P < 0.05). L. KEGG analysis of metabolites. M. Metabolite expression levels in NC, LIM, and quercetin intervention groups were analyzed by metabonomics. Blue line: glycolytic pathway; Green line: pentose phosphate pathway; Purple line: hexose branch.

Furthermore, WB and immunofluorescence results revealed that the expression levels of the glycolytic enzymes HK2, PFKL, PKM2, and LDHA were significantly elevated in the medium- and high-dose quercetin groups (45 mg/kg and 60 mg/kg) (all ∗P < 0.05 or ∗∗∗P < 0.001) (Fig. 3A–N‒W), along with increased mitochondrial membrane potential (all ∗P < 0.05 or ∗∗∗P < 0.001) (Fig. 4A and B), decreased reactive oxygen species levels (all ∗P < 0.05 or ∗∗∗P < 0.001) (Fig. 4C and D),and lactic acid levels (all ∗P < 0.05 or ∗∗∗P < 0.001) (Fig.4 E). The low-dose group (35 mg/kg) showed no significant changes.

To evaluate the metabolic effects of quercetin, we performed OCR and ECAR analyses using a Seahorse XFp Analyzer. In the myopic retina (LIM group), basal, maximal, and ATP-coupled mitochondrial oxygen consumption rates (OCR) were significantly decreased (all ∗P < 0.05 or ∗∗∗P < 0.001) (Fig. 4F–H). Conversely, Que-H and Que-M group successfully restored these OCR parameters (all ∗P < 0.05 or ∗∗∗P < 0.001) (Fig. 4G and H). Similarly, ECAR analysis revealed that basal glycolysis, induced glycolysis, and compensatory glycolysis were significantly lower in the LIM group than in the control group, while quercetin intervention effectively reversed these glycolytic deficits (all ∗P < 0.05 or ∗∗∗P < 0.001) (Fig. 4I–K). These functional data suggest that quercetin mitigates myopia progression by restoring retinal glycolysis and subsequently balancing oxidative phosphorylation (all ∗P < 0.05 or ∗∗∗P < 0.001) (Fig. 4J and K). Targeted metabolomics analyses were conducted to measure the concentrations of key glycolysis-related factors (Fig. 4L). In myopic retinas, the levels of l-lactic acid (LA) were reduced (all ∗P < 0.05), indicating decreased glycolysis in myopia, which is consistent with the in vivo enzyme activity results. KEGG pathway analysis revealed significant alterations in metabolism-related pathways, including glycolysis, oxidative phosphorylation, FOXO, and other signaling pathways, in myopia (Fig. 4M). Overall, these findings suggest that quercetin may mitigate the progression of myopia by modulating dysregulated glycolysis via the AKT/FOXO/HK2 axis.

### 3.4 Downregulation of glycolysis and upregulation of oxidative phosphorylation in retinal neurons are involved in myopia progression

#### 3.4.1 The effect of inhibiting glycolysis on myopia

**Fig. 5:**
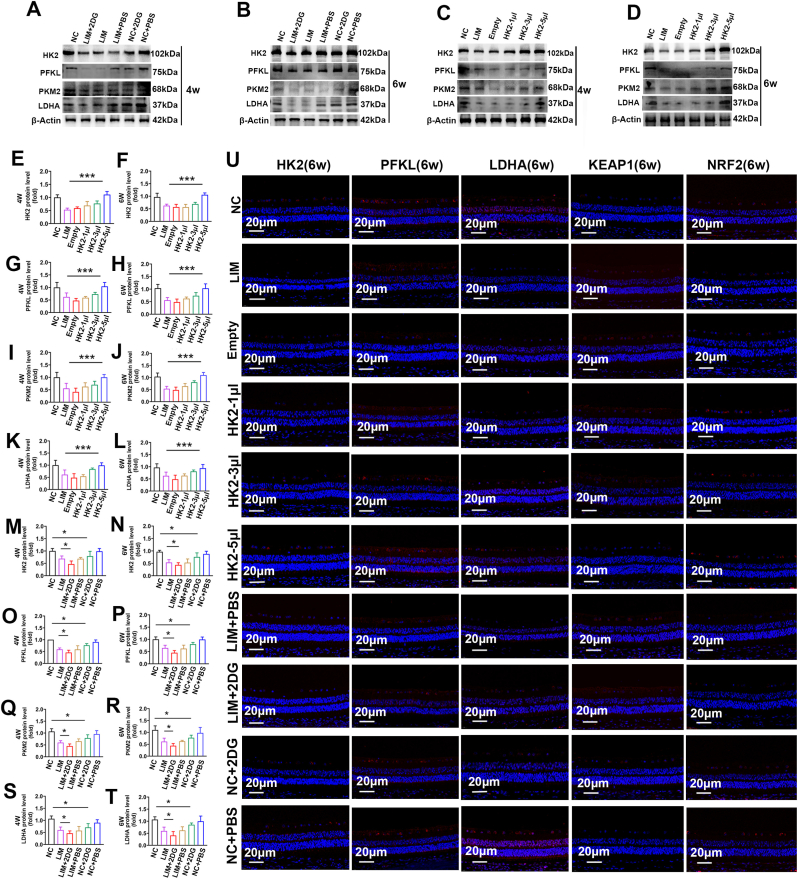
The influence of changes in glycolysis level on myopia. A-B. HK2, PFKL, PKM2, and LDHA expression detected by Western blot in NC, LIM, LIM+2DG, LIM + PBS, NC+2DG, NC + PBS group in 4w and 6w. Samples derived from the same experiment and that blots were processed in parallel. C-D. HK2, PFKL, PKM2, and LDHA expression detected by Western blot in NC, LIM, Empty, HK2-1μl, HK2-3μl, HK2-5μl, group in 4w and 6w. Samples derived from the same experiment and that blots were processed in parallel. E-L. Bar graphs of Western blot analysis for HK2, PFKL, PKM2, and LDHA in NC, LIM, Empty, HK2-1μl, HK2-3μl, HK2-5μl group in 4w and 6w. (∗∗∗P < 0.001, ∗P < 0.05). M-T. Bar graphs of Western blot analysis for HK2, PFKL, PKM2, and LDHA in NC, LIM, LIM+2DG, LIM + PBS, NC+2DG, NC + PBS group in 4w and 6w. (∗∗∗P < 0.001, ∗P < 0.05). U. HK2, PFKL, LDHA, NRF2 and Keap1 immunofluorescence staining in NC, LIM, Empty, HK2-1μl, HK2-3μl, HK2-5μl, LIM+2DG, LIM + PBS, NC+2DG, NC + PBS group in 6w.

To elucidate the mechanism underlying this metabolic shift during myopia development, we assessed whether a baseline reduction in retinal glycolysis induces myopia. Following 4 and 6 weeks of 2-DG intraperitoneal injection, the expression levels of HK2, PFKL, PKM2, and LDHA were significantly reduced (all ∗P < 0.05) (Fig. 5A, B, M-T, U), indicates a decrease in glycolysis level.

**Fig. 6:**
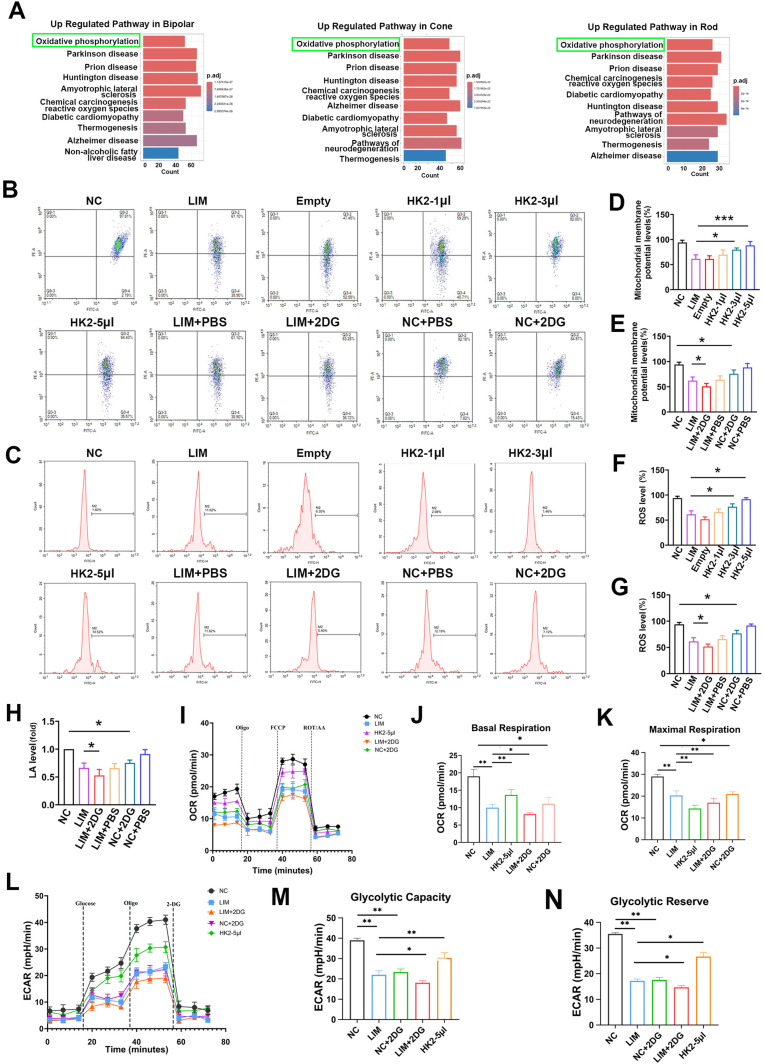
Glycolysis influences the effect of oxidative phosphorylation on myopia. A. Single-cell sequencing KEGG analysis. B. Mitochondrial membrane potential detection in NC, LIM, Empty, HK2-1μl, HK2-3μl, HK2-5μl, LIM+2DG, LIM + PBS, NC+2DG, NC + PBS groups at 6 weeks. C. ROS detection in NC, LIM, Empty, HK2-1μl, HK2-3μl, HK2-5μl, LIM+2DG, LIM + PBS, NC+2DG, NC + PBS groups at 6 weeks. D. Bar graphs of Mitochondrial membrane potential analysis in NC, LIM, Empty, HK2-1μl, HK2-3μl, HK2-5μl group (∗∗∗P < 0.001, ∗P < 0.05). E. Bar graphs of Mitochondrial membrane potential analysis in NC, LIM, LIM+2DG, LIM + PBS, NC+2DG, NC + PBS group (∗P < 0.05). F. Bar graphs of ROS analysis in NC, LIM, Empty, HK2-1μl, HK2-3μl, HK2-5μl group (∗P < 0.05). G. Bar graphs of ROS analysis in NC, LIM, LIM+2DG, LIM + PBS, NC+2DG, NC + PBS group (∗∗∗P < 0.001, ∗P < 0.05). H. Bar graphs of LA analysis in NC, LIM, LIM+2DG, LIM + PBS, NC+2DG, NC + PBS group (∗∗∗P < 0.001, ∗P < 0.05). I. Mitochondrial respiratory function in the retina after 6 weeks of myopia induction. J. Bar graphs of basal mitochondrial respiration analysis in NC, LIM, HK2-5 μl, LIM+2DG, NC+2DG (∗∗P < 0.01, ∗P < 0.05). K. Bar graphs of maximal mitochondrial respiration analysis in NC, LIM, HK2-5 μl, LIM+2DG, NC+2DG (∗∗P < 0.01). L. Glycolytic function in the retina after 6 weeks of myopia induction. M. Bar graphs of glycolytic capacity analysis in NC, LIM, HK2-5 μl, LIM+2DG, NC+2DG (∗∗P < 0.01). N. Bar graphs of glycolytic reserve analysis in NC, LIM, HK2-5 μl, LIM+2DG, NC+2DG (∗∗∗P < 0.001, ∗∗P < 0.01, ∗P < 0.05) NC: normal control, LIM: lens-induced myopia, Empty: (LIM + empty AAV vector), HK2-1μl: (LIM + AAV-HK2 1 μL intravitreal injection), HK2-3μl: (LIM + AAV-HK2 3 μL intravitreal injection), HK2-5μl: (LIM + AAV-HK2 5 μL intravitreal injection), LIM+2DG: (LIM + 2-deoxy-d-glucose 500 mg/kg intraperitoneal injection), LIM + PBS: (LIM + PBS intraperitoneal injection), NC+2DG: (NC + 2-deoxy-d-glucose 500 mg/kg intraperitoneal injection), NC + PBS: (NC + PBS intraperitoneal injection).

**Fig. 7:**
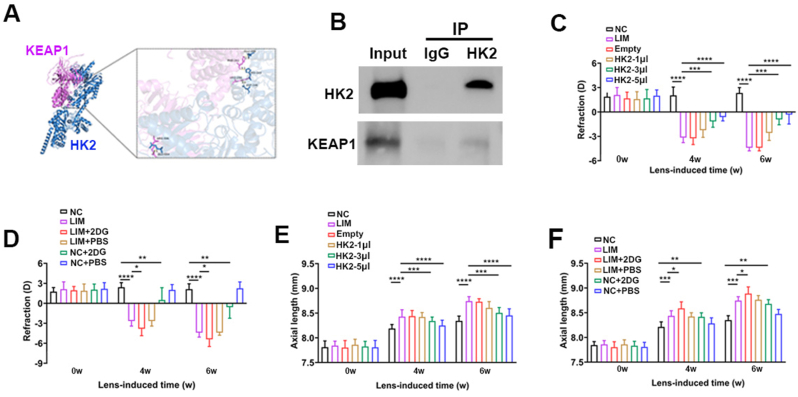
HK2 interacts with KEAP1 and regulates the development of myopia. A. KEAP1 and HK2 molecules docking. B. The protein interaction between KEAP1 and HK2 was analyzed by co-immunoprecipitation. C. The mean values of refraction in the right eyes of the guinea pigs after myopia induction for 0, 4, and 6 weeks between the NC, LIM, Empty, HK2-1μl, HK2-3μl, HK2-5μl groups (∗∗∗P < 0.001). D. The mean values of refraction in the right eyes of the guinea pigs after myopia induction for 0, 4, and 6 weeks between the NC, LIM, LIM+2DG, LIM + PBS, NC+2DG, NC + PBS groups ((∗∗P < 0.01, ∗P < 0.05)). E. The mean values of axial length in the right eyes of the guinea pigs after myopia induction for 0, 4, and 6 weeks between the NC, LIM, Empty, HK2-1μl, HK2-3μl, HK2-5μl groups (∗∗P < 0.01). F. The mean values of axial length in the right eyes of the guinea pigs after myopia induction for 0, 4, and 6 weeks between the NC, LIM, LIM+2DG, LIM + PBS, NC+2DG, NC + PBS groups (∗∗∗P < 0.001, ∗∗P < 0.01, ∗P < 0.05). ROS: Reactive oxygen species, NC: normal control, LIM: lens-induced myopia, Empty: (LIM + empty AAV vector), HK2-1μl: (LIM + AAV-HK2 1 μL intravitreal injection), HK2-3μl: (LIM + AAV-HK2 3 μL intravitreal injection), HK2-5μl: (LIM + AAV-HK2 5 μL intravitreal injection), LIM+2DG: (LIM + 2-deoxy-d-glucose 500 mg/kg intraperitoneal injection), LIM + PBS: (LIM + PBS intraperitoneal injection), NC+2DG: (NC + 2-deoxy-d-glucose 500 mg/kg intraperitoneal injection), NC + PBS: (NC + PBS intraperitoneal injection).

Furthermore, compared with the LIM group, the expression of NRF2 in the retina of the 2DG group decreased while the expression of Keap1 increased, both of which are established biomarkers of oxidative stress (Fig. 5U). Single-cell RNA sequencing and proteomic analysis revealed that myopic retinal neurons activation of the oxidative phosphorylation signaling pathway (Figs. 6A and 1H, L). Protein interaction predictions (KEAP1-HK2) and docking scores (−313.33) suggested a KEAP1–HK2 interaction (Fig. 7A), which was confirmed by Co-IP in guinea pig retinas (Fig. 7B). Additionally, we noted a significant reduction in the mitochondrial membrane potential (all ∗P < 0.05 or ∗∗∗P < 0.001) (Fig. 6B–D), elevated reactive oxygen species (all ∗P < 0.05 or ∗∗∗P < 0.001) (Fig. 6C–F), and reduced lactate levels (all ∗P < 0.05 or ∗∗∗P < 0.001) (Fig. 6H).

The regulatory role of 2DG-mediated glycolysis in retinal metabolism was further validated via Seahorse assays. The results demonstrated that the LIM group presented significantly decreased basal, maximal, and ATP-coupled OCR, alongside lower glycolytic capacity and reserve (all ∗P < 0.05 or ∗∗P < 0.01) (Fig. 6I–N). Following the inhibition of glycolysis via 2DG technology, mitochondrial oxygen consumption and glycolytic rates were significantly reduced in the retina of the 2DG intervention groups (LIM+2DG vs. LIM, NC+2DG vs. NC) (all ∗P < 0.05 or ∗∗P < 0.01) (Fig. 6J, K, M, N). These findings indicate that reduced glycolysis is accompanied by increased oxidative phosphorylation and oxidative stress.

Compared with NC, NC+2DG eyes presented significantly greater myopic shifts and axial length (AL) elongation (all ∗∗∗∗P < 0.0001 or ∗∗∗P < 0.001) (Fig. 7D–F). Similarly, compared with LIM eyes, LIM+2DG eyes presented significantly greater myopic shifts and ALs; nevertheless, although LIM eyes presented greater myopic shifts and ALs than did NC+2DG eyes, the difference was not statistically significant (Fig. 7D–F).

#### 3.4.2 The effect of HK2 overexpression on myopia

Next, we investigated whether the overexpression of HK2 and the restoration of retinal glycolysis could inhibit myopia progression. To systematically evaluate the restorative potential of HK2, we compared the AAV-HK2 groups (1 μL, 3 μL, and 5 μL) against both the standard LIM group and the Empty vector control group to account for any effects of the viral delivery process. Four and six weeks after AAV-HK2 injection, retinal protein levels of HK2, PFKL, PKM2, and LDHA were significantly higher in AAV-HK2-treated eyes than in LIM-treated eyes (∗∗∗P < 0.001) (Fig. 5C, D, E-L, V). This increase was accompanied by elevated NRF2 expression and reduced Keap1 expression (Fig. 5U), as well as increased mitochondrial membrane potential (all ∗P < 0.05) (Fig. 6B–E) and reduced reactive oxygen species levels (all ∗P < 0.05 or ∗∗∗P < 0.001) (Fig. 6C–G). In contrast, restoring HK2 expression via AAV-HK2 significantly increased both mitochondrial oxygen consumption and glycolytic rates in the myopic retina (all ∗P < 0.05 or ∗∗P < 0.01) (Fig. 6l–N). These findings provide robust functional evidence that restoring HK2-mediated glycolysis protects against the metabolic exhaustion and oxidative stress associated with myopia progression.

Compared with the LIM group, the AAV-HK2-5 μL and AAV-HK2-3 μL groups presented a significant reduction in myopic shifts and AL changes, whereas the AAV-HK2-1 μL group did not significantly differ (all ∗∗∗∗P < 0.0001 or ∗∗∗P < 0.001) (Fig. 7C–E). These findings support the proposed critical role of the AKT/FOXO/HK2 axis in the metabolic regulation of myopia, although the involvement of secondary pathways cannot be entirely excluded.

### 3.5 Quercetin inhibits retinal neuron damage and myopia progression

**Fig. 8:**
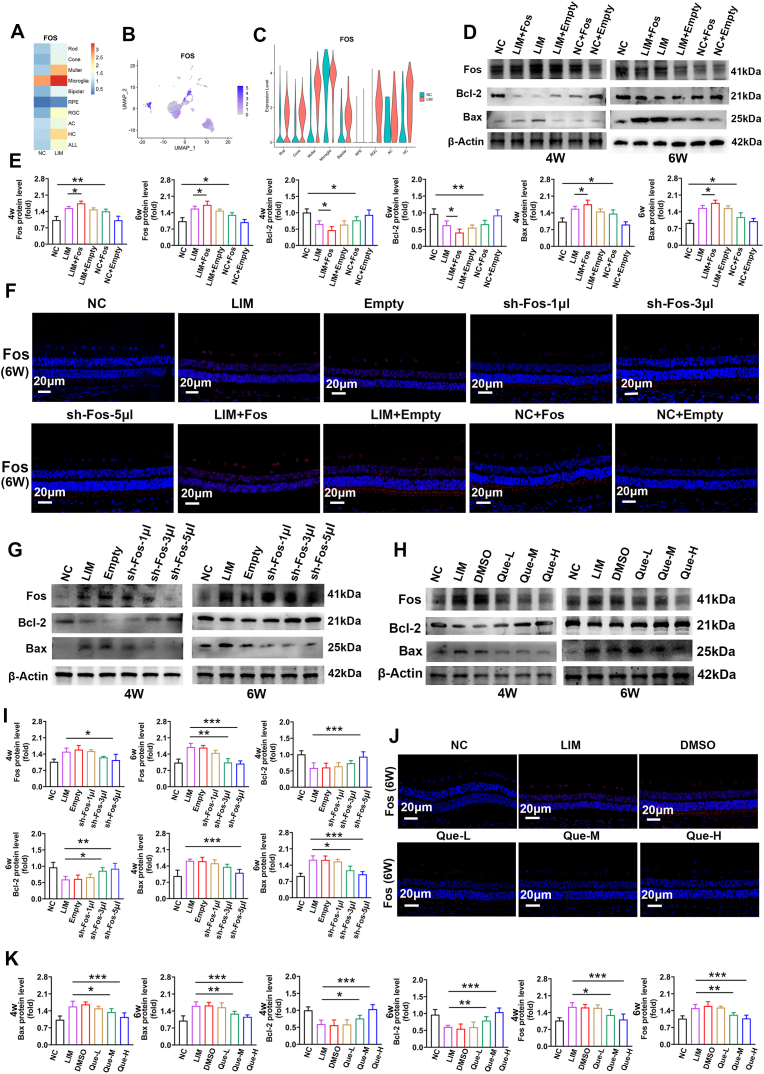
Effect of FOS expression on retinal apoptosis in myopia. A. The thermogram of Fos expression levels in various retinal cell types was analyzed by single-cell sequencing. B. The distribution of Fos in various retinal cell types was analyzed by single-cell sequencing. C. Fos expression in cell types. D. Fos, Bcl-2, and Bax expression detected by Western blot in NC, LIM, LIM + Fos, LIM + Empty, NC + Fos, NC + Empty group in 4w and 6w. Samples derived from the same experiment and that blots were processed in parallel. E. Bar graphs of Western blot analysis for Fos, Bcl-2, and Bax in NC, LIM, LIM + Fos, LIM + Empty, NC + Fos, NC + Empty group in 4w and 6w. (∗∗P < 0.01, ∗P < 0.05). F. Fos immunofluorescence staining in NC, LIM, empty, sh-Fos-1ul, sh-Fos-3ul, sh-Fos-5ul, LIM + Fos, LIM + Empty, NC + Fos, NC + Empty in 6w. G. Fos, Bcl-2, and Bax expression detected by Western blot in NC, LIM, empty, sh-Fos-1ul, sh-Fos-3ul, sh-Fos-5ul group in 4w and 6w. Samples derived from the same experiment and that blots were processed in parallel. H. Fos, Bcl-2, and Bax expression were detected by Western blot in NC, LIM, DMSO, Que-L, Que-M, and Que-H groups in 4w and 6w. Samples derived from the same experiment and that blots were processed in parallel. I. Bar graphs of Western blot analysis for Fos, Bcl-2, and Bax in NC, LIM, empty, sh-Fos-1ul, sh-Fos-3ul, sh-Fos-5ul group in 4w and 6w (∗∗∗P < 0.001, ∗∗P < 0.01, ∗P < 0.05). J. Fos immunofluorescence staining in NC, LIM, DMSO, Que-L, Que-M, Que-H group in 6w. K. Bar graphs of Western blot analysis for Fos, Bcl-2, and Bax in NC, LIM, DMSO, Que-L, Que-M, Que-H group in 4w and 6w (∗∗∗P < 0.001,∗∗P < 0.01, ∗P < 0.05).

Single-cell sequencing revealed increased Fos expression in myopic retinas across various cell types (rod, cone, Muller, bipolar, RGC, HC, and all) (P < 0.05, |log2FC| ≥ 0.25) (Fig. 8A–C, Table S1).

#### 3.5.1 The effect of fos overexpression on myopia

**Fig. 9:**
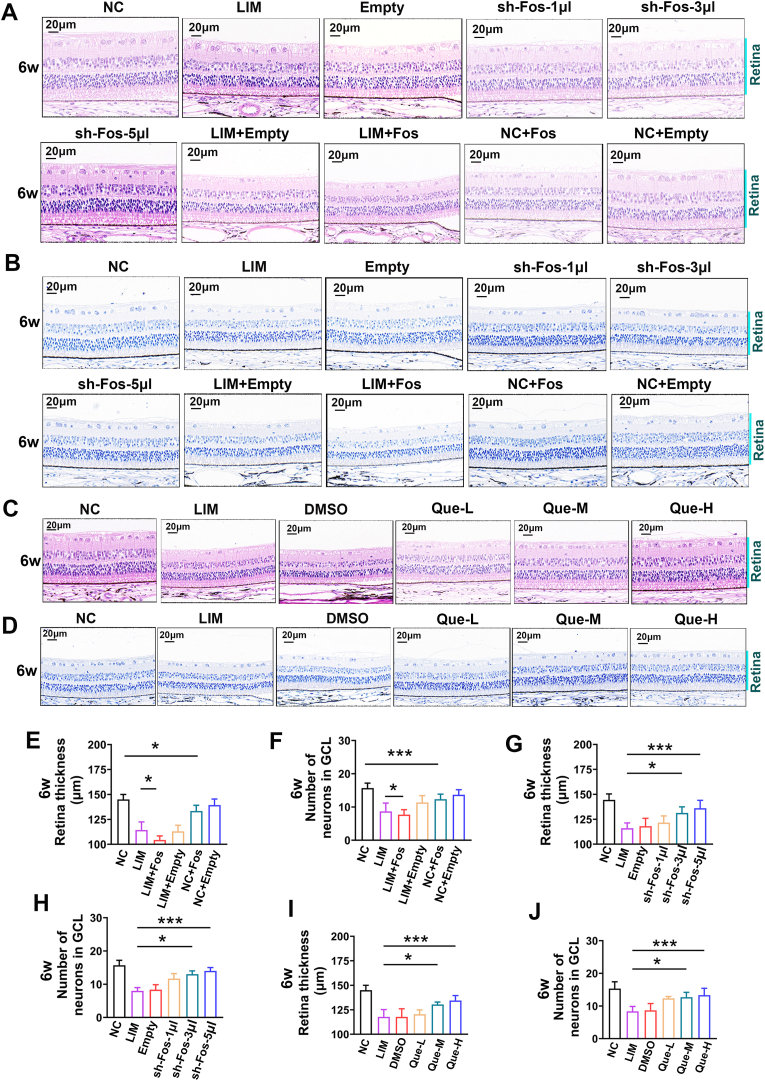
Effect of FOS expression on the structure of myopic optic network. A.The H&E staining of the retina in NC, LIM, empty, sh-Fos-1ul, sh-Fos-3ul, sh-Fos-5ul, LIM + Fos, LIM + Empty, NC + Fos, NC + Empty group in 6 weeks, sections marked in blue is the retina tissue. B. Retinal nissl staining in NC, LIM, empty, sh-Fos-1ul, sh-Fos-3ul, sh-Fos-5ul, LIM + Fos, LIM + Empty, NC + Fos, NC + Empty group in 6 weeks, sections marked in blue is the retina tissue. C. The H&E staining of the retina in NC, LIM, DMSO, Que-L, Que-M, Que-H group in 6 weeks, sections marked in blue is the retina tissue. D. Retinal nissl staining in NC, LIM, DMSO, Que-L, Que-M, Que-H group in 6 weeks, neuron particles were in dark blue. E. Bar graphs of H&E results of retinal thickness after myopic induction for 6 weeks in NC, LIM, LIM + Fos, LIM + Empty, NC + Fos, NC + Empty group (∗P < 0.05) F. Bar graphs of Nissl staining results of RGCs at 6 weeks after myopia induction in NC, LIM, LIM + Fos, LIM + Empty, NC + Fos, NC + Empty group (∗∗∗P < 0.001, ∗P < 0.05). G. Bar graphs of H&E results of retinal thickness after myopic induction for 6 weeks in NC, LIM, empty, sh-Fos-1ul, sh-Fos-3ul, sh-Fos-5ul group (∗∗∗P < 0.001). H. Bar graphs of Nissl staining results of RGCs at 6 weeks after myopia induction in NC, LIM, empty, sh-Fos-1ul, sh-Fos-3ul, sh-Fos-5ul group (∗∗∗P < 0.001, ∗P < 0.05). I. Bar graphs of H&E results of retinal thickness after myopic induction for 6 weeks in NC, LIM, DMSO, Que-L, Que-M, Que-H group (∗∗∗P < 0.001, ∗P < 0.05). J. Bar graphs of Nissl staining results of RGCs at 6 weeks after myopia induction in NC, LIM, DMSO, Que-L, Que-M, Que-H group (∗∗∗P < 0.001, ∗P < 0.05).

To elucidate the link between neuronal apoptosis and myopia development, we first assessed the impact of Fos overexpression. After AAV-Fos injection, the Fos and BAX levels were significantly elevated, whereas the BCL expression levels were significantly decreased (all ∗P < 0.05, ∗∗P < 0.01 or ∗∗∗P < 0.001) (Fig. 8D, E, F). Quantitative analysis of Western blot results revealed that AAV-Fos intervention led to a significant increase in the Bax/Bcl-2 ratio (approximately 3.5-fold increase compared to the NC group, P < 0.001), providing strong statistical support for Fos-driven apoptosis. This apoptotic shift was further confirmed by the observed 30-40% reduction in retinal thickness as measured in H&E sections (Fig. 9A–E), while retinal neuron Nissl staining also significantly decreased (all ∗P < 0.05 or ∗∗∗P < 0.001) (Fig. 9B–F).

**Fig. 10:**
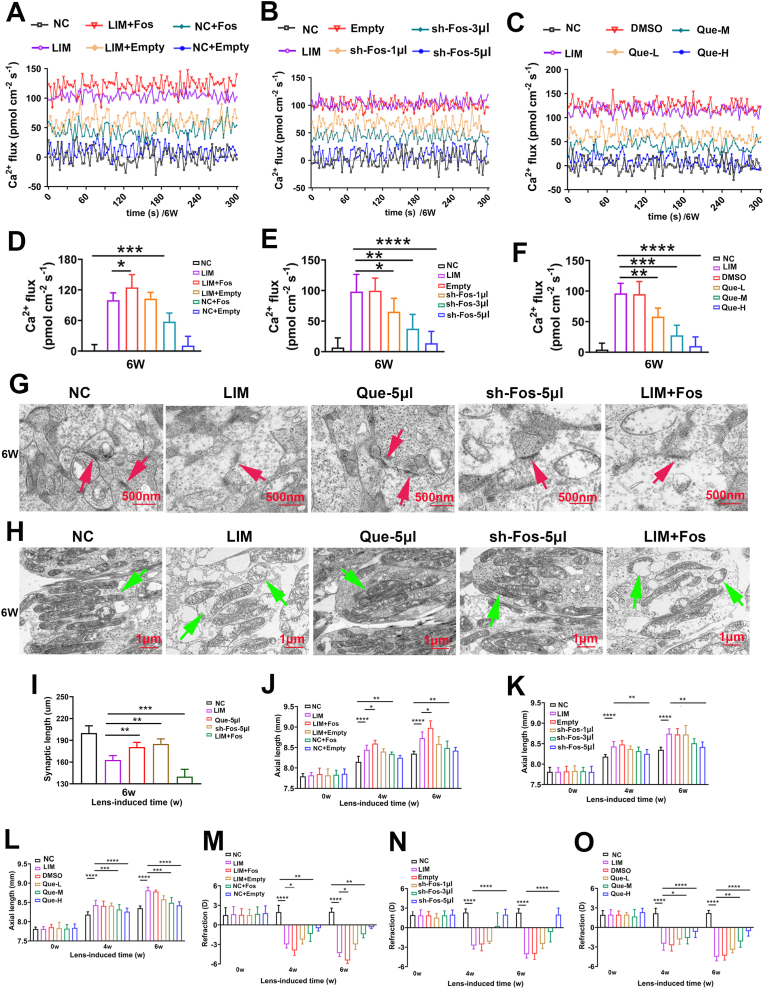
Effects of quercetin on retinal neurons in myopia. A. 5-Min data of Ca2+ (pmol·cm^−2^·s^−1^) in the retina recorded by non-invasive micro-test technology after myopic induction for 6 weeks in NC, LIM, LIM + Fos, LIM + Empty, NC + Fos, NC + Empty groups. B. 5-Min data of Ca2+ (pmol·cm^−2^·s^−1^) in the retina recorded by non-invasive micro-test technology after myopic induction for 6 weeks in NC, LIM, empty, sh-Fos-1ul, sh-Fos-3ul, sh-Fos-5ul groups. C. 5-Min data of Ca2+ (pmol·cm^−2^·s^−1^) in the retina recorded by non-invasive micro-test technology after myopic induction for 6 weeks in NC, LIM, DMSO, Que-L, Que-M, Que-H groups. D. Analysis of the retinal Ca^2+^ (pmol·cm^−2^·s^−1^) based on NMT for 6 weeks in NC, LIM, LIM + Fos, LIM + Empty, NC + Fos, NC + Empty groups (∗∗∗P < 0.001, ∗P < 0.05). E. Analysis of the retinal Ca^2+^ (pmol·cm^−2^·s^−1^) based on NMT for 6 weeks in NC, LIM, empty, sh-Fos-1ul, sh-Fos-3ul, sh-Fos-5ul groups (∗∗∗∗P < 0.0001. ∗∗P < 0.01). F. Analysis of the retinal Ca^2+^ (pmol·cm^−2^·s^−1^) based on NMT for 6 weeks in NC, LIM, DMSO, Que-L, Que-M, Que-H groups (∗∗∗∗P < 0.0001. ∗∗P < 0.01). G. The synapses of the NC, LIM, Que-H, sh-Fos-5ul, and LIM + Fos groups were detected by TEM. The red arrows indicate synapses. H. The mitochondria of the NC, LIM, Que-H, sh-Fos-5ul, and LIM + Fos groups were detected by TEM. The green arrows indicate mitochondria. I: The TEM was used to measure the synaptic lengths of the NC, LIM, Que-H, sh-Fos-5ul and LIM + Fos groups. J. The mean values of axial length in the right eyes of the guinea pigs after myopia induction for 0, 4, and 6 weeks between the NC, LIM, LIM + Fos, LIM + Empty, NC + Fos, NC + Empty groups (∗∗∗∗P < 0.0001, ∗∗∗P < 0.001). K. The mean values of axial length in the right eyes of the guinea pigs after myopia induction for 0, 4, and 6 weeks between the NC, LIM, empty, sh-Fos-1ul, sh-Fos-3ul, sh-Fos-5ul groups (∗∗∗∗P < 0.0001, ∗∗P < 0.01, ∗P < 0.05). L. The mean values of axial length in the right eyes of the guinea pigs after myopia induction for 0, 4, and 6 weeks between the NC, LIM, DMSO, Que-L, Que-M, Que-H groups (∗∗∗∗P < 0.0001, ∗∗∗P < 0.001). M. The mean values of refraction in the right eyes of the guinea pigs after myopia induction for 0, 4, and 6 weeks between the NC, LIM, LIM + Fos, LIM + Empty, NC + Fos, NC + Empty groups (∗∗∗∗P < 0.0001, ∗∗∗P < 0.001). N. The mean values of refraction in the right eyes of the guinea pigs after myopia induction for 0, 4, and 6 weeks between the NC, LIM, empty, sh-Fos-1ul, sh-Fos-3ul, sh-Fos-5ul groups (∗∗∗∗P < 0.0001, ∗P < 0.05). O. The mean values of refraction in the right eyes of the guinea pigs after myopia induction for 0, 4, and 6 weeks between the NC, LIM, DMSO, Que-L, Que-M, Que-H groups (∗∗∗∗P < 0.0001, ∗∗∗P < 0.001).

Specifically, after Fos overexpression, the pathological calcium efflux was significantly increased in the LIM + Fos group compared to the LIM group (all ∗∗P < 0.01 or ∗∗∗∗P < 0.0001) (Fig. 10A–D). This indicates impaired retinal neuronal function. Ultrastructural analysis further confirmed the causal role of Fos in mediating structural degradation. Electron microscopy analysis revealed aggravated synaptic damage (Fig. 10G) and mitochondrial damage (Fig. 10H) in the LIM and LIM + Fos groups compared with the NC group.

Compared with the NC group, the NC + Fos group presented significantly more severe myopia and longer axial lengths. Similarly, those from the LIM + Fos group presented significant greater diopter changes and AL values than did those from the LIM group (all ∗P < 0.05 or ∗∗P < 0.01) (Fig. 10I–L). However, while LIM eyes had greater myopic diopters and ALs than did NC + Fos eyes, the difference was not statistically significant (Fig. 10I–L). These results suggest that Fos overexpression exacerbates myopia development by promoting retinal apoptosis and altering the structure and function of retinal neurons.

#### 3.5.2 The effect of fos knockdown on myopia

Subsequently, we investigated whether Fos knockdown in the retina inhibits myopia development. In these assays, the Empty vector-injected LIM group served as the primary reference point to evaluate the specific efficacy of Fos reduction in counteracting myopic progression, ensuring that the therapeutic benefits were not attributed to the AAV vector itself. After 4 and 6 weeks of sh-Fos injection, retinal Fos and BAX expression levels were significantly lower in the sh-Fos 5 μL group than in the LIM group, whereas BCL expression levels were significantly elevated (all ∗P < 0.05, ∗∗P < 0.01 or ∗∗∗P < 0.001) (Fig. 8 F, G, I), an increase in retinal thickness (all ∗P < 0.05 or ∗∗∗P < 0.001) (Fig. 9A–G), and the recovery of retinal neuronal Nissl bodies (all ∗P < 0.05 or ∗∗∗P < 0.001) (Fig. 9B–H).

Specifically, Fos knockdown via sh-Fos significantly inhibited the pathological calcium efflux by approximately 55% in the sh-Fos 5 μL group compared to the LIM group (Fig. 10B–E, ∗∗∗∗P < 0.0001), indicating a substantial recovery of neuronal functional stability. Additionally, relatively intact synapses (Fig. 10 G) and mitochondrial structure (Fig. 10 H) were observed. This indicates that inhibiting FOS expression can restore functional and structural abnormalities in myopic retinal neurons.

Additionally, changes in myopic diopter and AL were significantly lower in the sh-Fos 5 μL group than in the LIM group (all ∗P < 0.05, ∗∗P < 0.01 or ∗∗∗∗P < 0.0001) (Fig. 10J–M).

#### 3.5.3 The effect of quercetin intervention on apoptosis of retinal neurons and myopia development

Compared with those in the LIM group, the retinal Fos and BAX expression levels in the quercetin intervention group were significantly lower, and the BCL expression levels were higher (all ∗P < 0.05, ∗∗P < 0.01 or ∗∗∗P < 0.001) (Fig. 8H–J, K). This intervention also increased retinal thickness (all ∗P < 0.05 or ∗∗∗P < 0.001) (Fig. 9C–I) and augmented retinal neuronal Nissl bodies (all ∗P < 0.05 or ∗∗∗P < 0.001) (Fig. 9D–J), resulting in decreased calcium ion outflow (all ∗P < 0.05 or ∗∗∗P < 0.001) (Fig. 10C–F). In the quercetin intervention group, electron microscopy revealed significant restoration of retinal synaptic (Fig. 10 G) and mitochondrial structures (Fig. 10 H) compared to the LIM group. This indicates that the intervention of quercetin can alleviate the functional and structural abnormalities of retinal neurons in myopia. Compared with those in the LIM group, the myopic diopter and AL in the quercetin intervention group were significantly lower (all ∗∗∗∗P < 0.0001 or ∗∗∗P < 0.001) (Fig. 10K–N).

These findings demonstrate that the Fos-induced increase in retinal cell apoptosis, along with damage to neuronal synapses and mitochondria, promotes the onset and progression of myopia. Quercetin treatment was associated with delayed myopia progression and reduced Fos expression, suggesting a potential link between these effects.

## 4 Discussion

Quercetin, a flavonoid compound found in fruits, vegetables, and other edible plants, has notable pharmacological effects and therapeutic potential. It acts as an inhibitor of the PI3K signaling pathway [19] and has neuroprotective properties by mitigating oxidative stress-induced neuronal damage [20,21]. In previous study, our research group found that phellendiol inhibits myopia progression by suppressing oxidative stress, but the specific mechanism remains unclear [22]. In this study, we provide evidence that quercetin may restores the glycolytic level in the retina by regulating the AKT/FOXO/HK2 axis, thereby slowing down the progression of myopia. Specifically, through single-cell RNA sequencing and proteomics analysis, we found that in the myopia model, the glycolysis in the retina significantly decreased. After quercetin treatment, the expressions of key glycolytic enzymes such as HK2, PFKL, and PKM2 recovered, and the lactate level significantly increased. These data further indicate that quercetin can regulate oxidative phosphorylation by restoring glycolysis and alleviate oxidative stress in the retina, thereby slowing down the progression of myopia. Our data provide concrete evidence for this mechanism; Western blot analysis demonstrated that medium and high doses of Quercetin (45-60 mg/kg) significantly downregulated the expression of PIK3Ca and AKT1 while simultaneously increasing *p*-FOXO3a levels. Crucially, this molecular shift directly correlated with the functional restoration of retinal metabolism, as evidenced by the Seahorse XFp analysis showing a significant increase in both basal glycolysis and compensatory glycolysis (Fig. 4I–K). Given that scRNA-seq revealed coordinated glycolytic suppression across multiple retinal cell types, Seahorse assays were performed on heterogeneous retinal single-cell suspensions to capture global tissue-level metabolic reprogramming. These findings suggest that quercetin may exert its effects, at least in part, by modulating the metabolic landscape of the myopic retina through the AKT/FOXO/HK2 signaling cascade, thereby providing a metabolic basis for its protective effects against myopia.

We acknowledge that definitive proof of pathway specificity would require experiments using AKT-specific inhibitors (e.g., MK-2206) or HK2 knockdown in the context of quercetin treatment. Such studies, while beyond the scope of the current investigation, represent important future directions to establish whether quercetin's protective effects are strictly dependent on this pathway or involve parallel antioxidant mechanisms.

And, we acknowledge that our protein interaction data were obtained through co-immunoprecipitation and molecular docking methods. Molecular docking is merely a predictive approach, but no dynamic in vivo validation was conducted. However, Co-IP remains the gold standard for detecting endogenous protein complexes, and independent studies have validated the AKT1-HK2 interaction using orthogonal methods [23,24]. Regarding quercetin's direct binding, our docking predictions are supported by multiple independent in silico studies [[25], [26], [27]], but definitive proof awaits techniques such as DARTS or CETSA. Despite these limitations, the convergence of our multi-omics data and functional rescue experiments provides robust support for our model.

As the metabolic “steward” of the retina, Müller cells not only maintain energy homeostasis by storing glycogen but also supply neurons with critical metabolic fuels (such as lactate) through aerobic glycolysis [28].

Vohra et al. showed that lactate is secreted from Müller cells and that RGCs preferentially utilize lactate over glucose as an energy source, with exogenous lactate significantly increasing RGC survival during glucose deprivation [29]. This research establishes a metabolic network wherein Müller cells function as the primary “glycolytic powerhouses,” supplying lactate to fuel adjacent neurons [[22], [23], [24], [25], [26], [27], [28], [29], [30]].

Müller cells primarily generate energy through glycolysis [30], which not only supports their function but also protects the blood‒retinal barrier and retinal neurons [31]. Muller cells are glial cells in the human retina that are essential for retinal support, regulate glutamate stability, metabolize glycogen, supply nutrients to retinal neurons, and produce antioxidants and neurotrophic factors [32]. Thus, the metabolic coupling between Müller cells and neurons—particularly RGCs—is fundamental to retinal health, and disruption of this support system compromises neuronal survival [33,34].

In this study, our single-cell RNA sequencing data (Fig. 1A–C) reveal that myopia disrupts this established metabolic network at two distinct levels. First, Müller cells exhibit significant downregulation of glycolytic enzymes (HK2, PFKL), indicating impaired lactate production and reduced metabolic support to neurons. Second, RGCs themselves show intrinsic suppression of glycolytic enzymes, compromising their ability to utilize available fuels. This finding aligns with recent work by Takahashi et al., who demonstrated that ENO1 dysfunction leads to glycolytic attenuation in RGCs, resulting in excessive dependence on OXPHOS under oxidative stress and contributing to excitotoxicity-induced RGC death [35].

As the final step in visual information processing, RGCs transmit electrical signals to relay cells in the lateral geniculate nucleus of the brain, suggesting that oxidative damage to RGCs may contribute significantly to myopia progression. Our experimental results show that in the myopia model, the glycolytic level of RGCs significantly decreases, while the oxidative phosphorylation level significantly increases, which is closely related to the elevated oxidative stress in the retina. The susceptibility of RGCs to this metabolic transition is further highlighted by our single-cell RNA sequencing data, which identified a distinct reduction in HK2 and PFKL transcripts specifically within the RGC cluster (Fig. 1A–E, F). This downregulation of glycolytic enzymes was accompanied by a concomitant activation of oxidative phosphorylation pathways as revealed by our proteomic and phosphoproteomic analysis (Fig. 1H–K). Moreover, the experimental data also indicate that the expressions of key enzymes for glycolysis in the retina, such as HK2, PFKL, and PKM2, significantly decrease during myopia progression, while the expressions of enzymes related to oxidative phosphorylation increase. Furthermore, our finding that retinal lactate (LA) and lactate dehydrogenase (LDHA) levels were significantly reduced in LIM-OD eyes (Fig. 1X and Y) reinforces the idea that RGCs undergo a state of ‘metabolic exhaustion’ during myopia progression.

This “dual-hit” disruption—affecting both the glial “supply side” (Müller cells) and the neuronal “demand side” (RGCs)-forces RGCs to upregulate compensatory oxidative phosphorylation, resulting in elevated ROS production and oxidative damage (Fig. 4, Fig. 6). This integrated model explains why the metabolic dysfunction in myopic retina is so severe: the lactate shuttle is compromised at both its source (Müller cells) and its target RGCs.

Beyond RGCs and Müller cells, other retinal cell types exhibit specialized metabolic profiles. Photoreceptors maintain high glycolytic flux despite abundant mitochondria, a configuration that protects against oxidative damage [36,37]. The retinal pigment epithelium (RPE) utilizes lactate produced by photoreceptors as an energy source, and disruption of this metabolic coupling contributes to retinal degeneration [38]. Aït-Ali et al. demonstrated that rod-derived cone viability factor (RdCVF) promotes cone survival specifically by stimulating aerobic glycolysis through interaction with Basigin-1 and GLUT1, providing a direct mechanistic link between glycolysis and neuronal survival [39].

This cell type-specific metabolic organization has important therapeutic implications: interventions targeting glycolysis must consider the distinct roles of each cell type. Enhancing glycolysis in Müller cells or RGCs (as with quercetin in our study) may be protective, whereas indiscriminately modulating glycolysis could have cell type-specific effects.

A previous study has reported conflicting roles of glycolysis in myopia, with some showing increased glycolysis in the sclera [40] and others (including ours) showing decreased glycolysis in the retina. This apparent contradiction reflects the fundamental difference in metabolic function between these tissues. The sclera is a connective tissue composed of fibroblasts that require glycolysis for anabolic support of ECM remodeling, a process essential for axial elongation. In contrast, the retina is neural tissue where glycolysis serves primarily as a protective mechanism against oxidative damage. Therefore, glycolysis modulation has opposite functional consequences in these tissues: enhancing scleral glycolysis promotes myopia [40], while enhancing retinal glycolysis (as with quercetin or HK2 overexpression in our study) inhibits myopia. This tissue-specific perspective resolves the apparent contradiction and underscores the need for targeted therapeutic strategies.

The apparent paradox of reduced lactate levels alongside enhanced oxidative phosphorylation in myopic retinas is resolved by understanding retinal metabolic compartmentalization. In the healthy retina, lactate is produced primarily by Müller cells via glycolysis and shuttled to neurons as their preferred oxidative fuel [[41], [42], [43]]. Thus, lactate concentration reflects glial glycolytic output, not neuronal OXPHOS rate. In our myopic retinas, reduced lactate (Fig. 1Y) coincides with downregulation of glycolytic enzymes in Müller cells (Fig. 1A–E-G), indicating a failure on the “supply side” of the lactate shuttle. When glia-derived lactate becomes scarce, neurons compensate by upregulating OXPHOS using alternative substrates such as fatty acids [44], explaining the co-existence of low lactate and high OXPHOS. This compensatory shift comes at a cost: increased ROS production and oxidative stress (Fig. 4, Fig. 6). Thus, low lactate reflects diminished glial supply, not increased neuronal consumption.

Neurons require protective mechanisms to prevent damage from OXPHOS while meeting their energy demands. Aerobic glycolysis, the primary mode of glucose metabolism in neurons, helps mitigate oxidative damage [3]. Unlike OXPHOS, aerobic glycolysis does not produce ROS, although its energy yield is only 1/18 that of OXPHOS. Studies have shown that lactate produced by astrocytes through increased glycolysis is absorbed by neurons as a major energy source, suggesting that neurons upregulate glycolysis under stimulation [45]. In this study, we observed that quercetin effectively inhibited the accumulation of oxidative stress by enhancing the level of glycolysis, and significantly slowed down the damage to retinal neurons. This indicates that quercetin, by regulating the balance between glycolysis and oxidative phosphorylation, may become a promising strategy for treating myopia. Our results are consistent with similar mechanisms in diabetic retinopathy (DR), suggesting that increasing glycolytic activity can reduce ROS production and improve the survival rate of retinal cells [46]. In retinitis pigmentosa, enhancing glycolysis through increased enolase 1 activity slows retinal degeneration [47]. Additionally, ROS have been implicated in various retinal injuries, including glaucoma, nonarteritic ischemic anterior neuropathy, traumatic optic neuropathy, and Leber hereditary optic neuropathy [48]. Our results directly support this link between glycolytic inhibition and oxidative damage. Specifically, when retinal glycolysis was pharmacologically inhibited by 2-DG, we observed a significant downregulation of HK2, PFKL, and PKM2 (Fig. 5U), which was coupled with a marked increase in ROS production and a significant reduction in mitochondrial membrane potential (Fig. 6C–F, G). Conversely, restoring glycolysis via AAV-HK2 overexpression successfully reversed these pathological changes (Fig. 6B–E), providing robust functional evidence that maintaining the glycolytic flux is essential for protecting retinal neurons against myopia-induced oxidative stress. Although systemic administration of 2-DG may exert peripheral metabolic effects, the intraocular rescue experiments strongly suggest that the exacerbation of myopia is primarily driven by local retinal glycolytic impairment rather than systemic toxicity. Vision development involves the generation of visual nerve impulses in the retina transmitted through three neuronal layers: photoreceptor cells, bipolar cells, and ganglion cells. In our study, we found that oxidative stress during the process of myopia not only affects photoreceptors but also includes retinal ganglion cells and other types of neurons. This further confirms the imbalance between glycolysis and oxidative phosphorylation, which plays a significant role in the damage of retinal neurons. Particularly in RGCs, the increase in oxidative phosphorylation is directly correlated with the elevated level of oxidative stress, providing new clues for the treatment of myopia. By regulating the level of glycolysis, we may be able to reduce the nerve damage caused by oxidative phosphorylation, thereby inhibiting the progression of myopia.

In summary, this study revealed the metabolic reprogramming between glycolysis and oxidative phosphorylation during the process of myopia, especially the changes in retinal ganglion cells. Quercetin demonstrates potential in the treatment of myopia by restoring glycolysis and reducing oxidative stress.

Visual information is transmitted along the visual pathway to the brain, forming the visual process. Fos, initially identified as a proto-oncogene associated with bone tumors, has since been recognized as a marker of brain activity, particularly after exposure to harmful stimuli such as seizures [49]. Fos-related proteins are implicated in the postsynaptic response to amino acid neurotransmitters in various amacrine and ganglion cells [50]. Fos activation is linked to retinal apoptosis, with increased expression observed during light-induced retinal cell death [[51], [52], [53], [54]]. In animal models, Fos contributes to photoreceptor cell death: rod photoreceptors in wild-type mice undergo apoptosis upon exposure to white light, whereas Fos knockout mice are protected [53,54]. In this study, we further verified the role of Fos in the progression of myopia. We observed that the apoptosis of retinal ganglion cells and other retinal cells significantly increased, accompanied by the upregulation of Fos expression. Especially in the LIM-induced myopia model, the expression of Fos in retinal RGCs and photoreceptors significantly increased, and this change was directly related to the increase in neuronal apoptosis. The observation that Fos overexpression alone (NC + Fos) did not significantly exacerbate myopia progression, despite inducing apoptosis markers, suggests that Fos functions as a ‘stress amplifier’ rather than a standalone initiator. The pre-existing metabolic dysfunction in the LIM retina, characterized by suppressed glycolysis and elevated oxidative stress, provides a permissive environment that amplifies Fos-mediated apoptosis. This ‘two-hit’ model, wherein metabolic vulnerability (first hit) and pro-apoptotic signaling (second hit) synergize to drive pathology, is consistent with established concepts in neurodegeneration and with the known role of Fos as a sensor of visual environment changes. Literature shows that oxidative stress and intracellular calcium overload, resulting from impaired glycolysis and compensatory OXPHOS upregulation, strongly induce immediate early genes like Fos via MAPK/ERK and CREB activation [[55], [56], [57]]. In myopic retinas, impaired glycolysis (due to AKT/FOXO-mediated HK2 suppression) makes neurons rely on OXPHOS, elevating ROS production and disrupting calcium homeostasis. This pro-oxidative environment activates the AP-1 complex, with Fos as a core component. Once induced, Fos amplifies apoptosis (increased Bax/Bcl-2 ratio), causing synaptic degeneration and mitochondrial damage. Critically, quercetin intervenes at the cascade's apex: by restoring glycolysis via AKT/FOXO/HK2 modulation, it reduces oxidative stress, indirectly suppressing Fos activation and its apoptotic consequences. Thus, Fos acts as a metabolic stress-responsive effector, not an independent driver, explaining why HK2 overexpression and Fos knockdown yield similar therapeutic effects. The experimental results indicated that the expression of Fos significantly increased in the early stage of myopia and was closely related to the death of retinal neurons. We also found that the expression of Fos in RGCs was the most significant, and it was positively correlated with the apoptotic level of retinal ganglion cells, further demonstrating the crucial role of Fos in retinal neuron damage.

In particular, during the myopia induction process in our experiment, the apoptosis of RGCs and the increase in Fos expression occurred simultaneously, suggesting that Fos may play an important role in the death of retinal neurons in myopia. By targeting the expression of Fos, we can effectively reduce the apoptosis of retinal ganglion cells and significantly slow down the progression of myopia. This finding supports the view that Fos is a potential target for treating myopia. The causal role of Fos in mediating structural degradation is further substantiated by our ultrastructural analysis. Transmission electron microscopy (TEM) revealed that Fos overexpression induced severe mitochondrial swelling and synaptic damage in retinal neurons (Fig. 10G, H, I). Importantly, inhibiting Fos expression—either through sh-Fos or quercetin intervention—effectively preserved mitochondrial integrity and synapse length (Fig. 10G, H, I). These structural improvements coincide with the reduction in calcium ion outflow observed via Non-invasive Micro-test Technology (Fig. 10C–F), reinforcing that Fos inhibition preserves retinal neuronal function by maintaining cellular homeostasis and mitochondrial health. It is worth noting that although the upregulation of Fos is usually associated with neuronal damage and death, we found that in the condition of Fos inhibition, the progression of myopia was significantly slowed down, and the survival rate of retinal neurons was significantly increased, providing important experimental evidence for Fos inhibition therapy.

Based on the results of this study, we believe that the excessive expression of Fos may be one of the important reasons for retinal neuron damage in myopia, especially the apoptosis of RGCs. By targeting the expression of Fos and its related pathways, it may provide a new intervention strategy for slowing down the progression of myopia. In future research, exploring the role of Fos in other retinal neuron types and its relationship with oxidative stress and metabolic reprogramming will further improve our understanding of the role of Fos in the pathogenesis of myopia and provide more targeted therapeutic approaches for clinical treatment (Fig. 10).

## 5 Conclusion

**Fig. 11:**
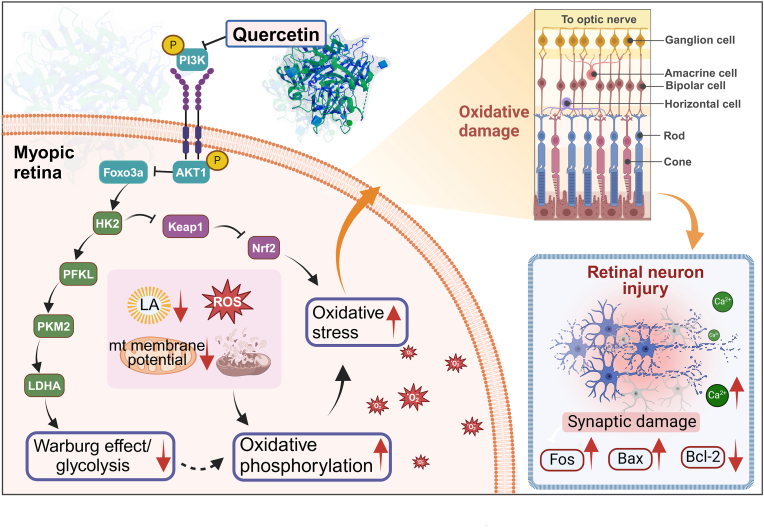
Quercetin restores glycolysis by regulating the AKT/FOXO/HK2 axis, thereby reducing oxidative phosphorylation and oxidative stress, and alleviating retinal neuron damage and apoptosis, thereby alleviating the development of myopia.

Multiomics and molecular analyses suggest that quercetin modulates myopic glycolysis, and our data highlight the AKT/FOXO/HK2 signaling pathway as a potential mechanism for this regulation. We also demonstrated that in myopia, the degree of glycolysis in neuronal cells decreases, oxidative phosphorylation increases, and the occurrence of oxidative stress promotes the progression of myopia. Furthermore, our research demonstrated that inhibiting Fos expression in the myopic retina can reduce neuronal cell injury and apoptosis, potentially slowing down myopia progression. Collectively, our findings suggest that quercetin may attenuate myopia progression, at least in part, by modulating the AKT/FOXO/HK2 axis to restore retinal glycolysis, thereby reducing oxidative stress and protecting against neuronal damage(Fig. 11). However, given the multi-target nature of quercetin, the contribution of other antioxidant pathways cannot be excluded. This study provides an experimental basis for understanding the pathogenesis of myopia and identifies the AKT/FOXO/HK2 axis as a potential therapeutic target for intervention strategies in treating myopia.

## Patient consent for publication

Not Applicable.

## Use of artificial intelligence tools

Not Applicable.

## Ethics approval and consent to participate

The present study was approved by the Experimental Animal Ethics Review Committee of the Affiliated Hospital of Shandong University of Traditional Chinese Medicine (Approval number: AWE-2022-055) and strictly followed the principles of the Statement of Animals Research in Vision and Ophthalmic (ARVO).

## CRediT authorship contribution statement

**Ruixue Zhang:** Conceptualization, Data curation, Investigation, Methodology, Writing – original draft. **Miao Zhang:** Data curation. **Yunxiao Xie:** Data curation. **Huixia Wei:** Investigation. **Zhaohui Yang:** Supervision. **Ying Wen:** Validation. **Jiawen Hao:** Supervision. **Yongle Du:** Supervision. **Yuanting Yang:** Supervision. **Xuewei Yin:** Validation. **Yinqiao Zhang:** Supervision. **Wenjun Jiang:** Validation. **Hongsheng Bi:** Supervision. **Dadong Guo:** Methodology, Visualization, Writing – original draft.

## Declaration of competing interest

The authors declare that they have no known competing financial interests or personal relationships that could have appeared to influence the work reported in this paper.

## Figures and Tables

**Figure:**
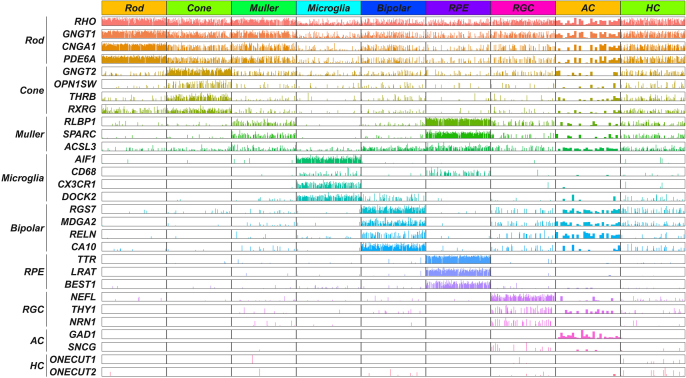
Fig.S1 Differential gene expression confirmed the identification of cell marker genes for 9 assigned cell types in the retina. The color bar contains the name and number of the core cell that represents the type.

**Figure:**
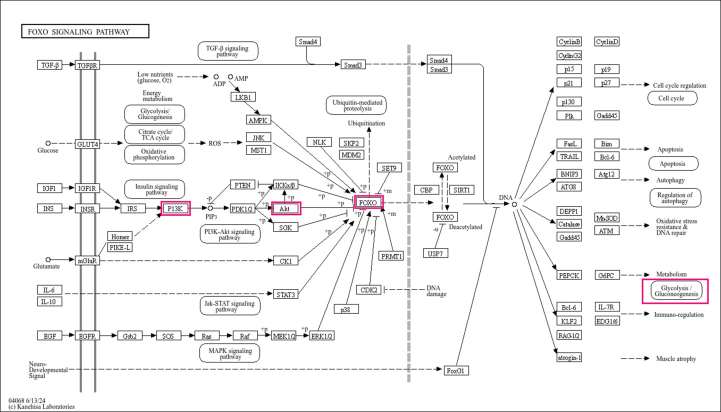
Fig.S2 FOXO signaling pathway map downloaded from KEGG website(www.genome.jp).

**Figure:**
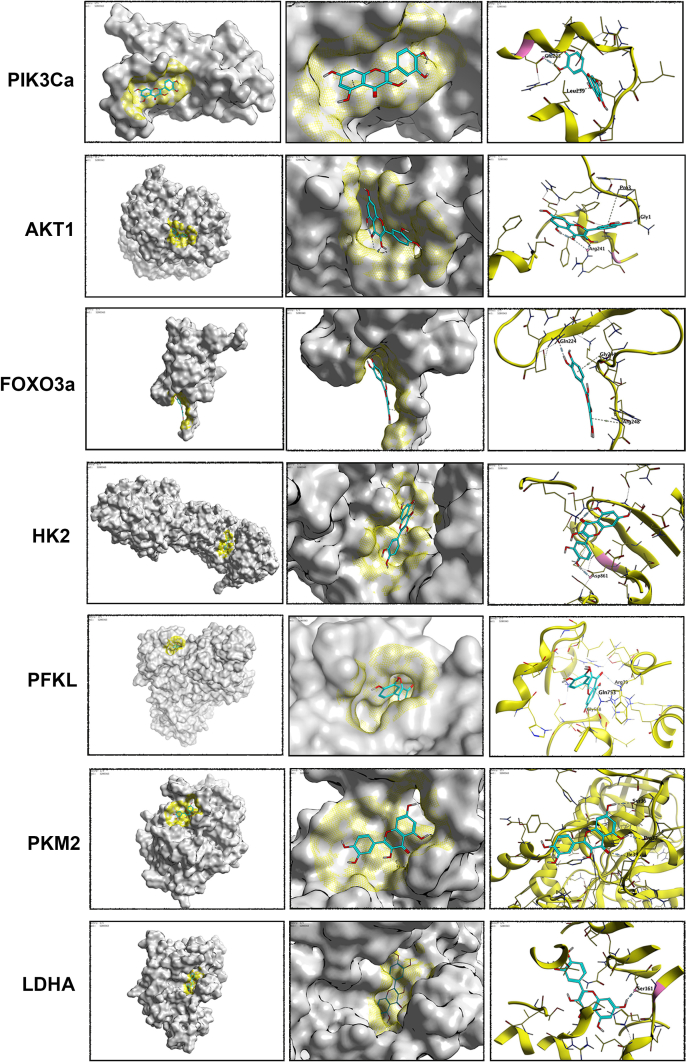
Fig.S3 Quercetin regulates molecular docking of PI3KCa, AKT1, FOXO3a, HK2, PFKL, PKM2, and LDHA.

## Data Availability

Data will be made available on request.

## Abbreviations

2DG: 2-deoxyglucose
AAV: Adeno-associated virus
NMT: Non-invasive micro-test technology
HE: Hematoxylin&eosin
PI3K: Phosphoinositide 3-kinase
HK2: Hexokinase 2
LIM: Lens-induced myopia
NC: Normal control
DMSO: Dimethyl sulfoxide
scRNA-seq: Single-cell RNA sequencing
PIK3CA: Phosphatidylinositol-4,5-bisphosphate 3-kinase, catalytic subunit alpha
AKT1: AKT Serine/Threonine Kinase 1
FOXO3a: Forkhead box O3
PFKL: Phosphofructokinase, Liver Type
PKM2: Pyruvate kinase isozyme typeM2
LDHA: l-lactate dehydrogenase
WT: Wild-type
MT: Mutant
ROS: Reactive oxygen species
LD: Lactate dehydrogenase
UHPLC-MS: Ultra-high performance liquid chromatography mass spectrometry
LC: Liquid chromatography
co-IP: Coimmunoprecipitation
SDS-PAGE: Sodium Dodecyl Sulfate PolyAcrylamide Gel Electrophoresis
NMT: Non-invasive Micro-test Technology
BSA: Bovine albumin
DAPI: 4′,6-diamidino-2-phenylindole
DAVID: Database for Annotation, Visualization, and Integrated Discovery
WB: Western blot
KEGG: Kyoto Encyclopedia of Genes and Genomes
G1P: d-glucose-1-phosphate
G6P: d-glucose-6-phosphate
F6P: d-fructose-6-phosphate
Glu: d-glucose
Fru: Fructose
FBP: d-fructose-1,6-diphosphate
GAP: glyceraldehyde 3-phosphate
2,3-DPG: 2,3-diphosphoglycerate
DHAP: Dihydroxyacetone phosphate
3 PG: 3-phosphoglyceric acid
2 PG: 2-phosphoglyceric acid
PEP: phosphoenolpyruvate
PA: Pyruvic acid
LA: l-lactic acid
X5P: d-xylose-5-phosphate
Ru5P: d-ribulose-5-phosphate
R5P: d-ribose-5-phosphate
S7P: Sedoheptulose-7-phosphate
6 PG: D-6-phosphogluconic acid
Keap1: Kelch-like ECH-associated protein 1
NRF2: Nuclearrespiratoty factor 2
AL: Axial length
RPE: Retinal pigment epithelial
ShRNA: Short hairpin RNA
BCL: B-cell lymphoma
RGCs: Retinal ganglion cells
OXPHOS: Oxidative phosphorylation
DR: Diabetic retinopathy
