# Supplementary material for: Quercetin improves retinal glycolysis to slow myopia progression through orchestrating the AKT/FOXO/HK2 axis
Source: Redox Biol. 2026 Mar 31;93:104139. doi: 10.1016/j.redox.2026.104139 (PMC13092674; doi:10.1016/j.redox.2026.104139)
Supplement: Multimedia component 1 [file mmc1.docx]

**Table S1 Single-cell RNA sequencing was performed to profile the transcript levels of HK2, PKM and PFKL across distinct cell populations.**

| **Cell Type** | **Gene** | **p_val** | **p_val_adj**  **(FDR)** | **avg_log2FC** | **pct.1**  **（NC）** | **pct.2**  **（LIM）** |
| --- | --- | --- | --- | --- | --- | --- |
| Bipolar | HK2 | 8.90E-05 | 1.00E+00 | -0.441 | 0.647 | 0.740 |
| Cone | HK2 | 1.10E-07 | 1.87E-03 | -0.399 | 0.913 | 0.832 |
| HC | PFKL | 4.73E-04 | 1.00E+00 | -0.377 | 0.665 | 0.306 |
| Microglia | PKM | 2.66E-01 | 1.00E+00 | -0.310 | 0.825 | 0.671 |
| Muller | HK2 | 1.46E-05 | 2.47E-01 | -0.445 | 0.279 | 0.139 |
| Muller | PFKL | 5.62E-11 | 9.54E-07 | -0.297 | 0.495 | 0.221 |
| Muller | PKM | 2.63E-01 | 1.00E+00 | -0.477 | 0.777 | 0.653 |
| RGC | PFKL | 1.74E-01 | 1.00E+00 | -1.154 | 0.324 | 0.342 |
