# Supplementary material for: Quercetin improves retinal glycolysis to slow myopia progression through orchestrating the AKT/FOXO/HK2 axis
Source: Redox Biol. 2026 Mar 31;93:104139. doi: 10.1016/j.redox.2026.104139 (PMC13092674; doi:10.1016/j.redox.2026.104139)
Supplement: Multimedia component 2 [file mmc2.docx]

Table S2. Prediction of FOXO3 and HK2 binding sites at ASPAR sites.

| **Matrix ID** | **Name** | **Score** | **Relative score** | **Sequence ID** | **Start** | **End** | **Strand** | **Predicted sequence** |
| --- | --- | --- | --- | --- | --- | --- | --- | --- |
| [**MA0157.4**](https://jaspar.elixir.no/matrix/MA0157.4) | MA0157.4.Foxo3 | 12.857997 | 0.9999999915029477 | Hk2 | 858 | 864 | - | GTAAACA |
| [**MA0157.1**](https://jaspar.elixir.no/matrix/MA0157.1) | MA0157.1.FOXO3 | 11.549052 | 0.9800807642952656 | Hk2 | 858 | 865 | - | GGTAAACA |
| [**MA0157.3**](https://jaspar.elixir.no/matrix/MA0157.3) | MA0157.3.Foxo3 | 12.51916 | 0.9512392120542189 | Hk2 | 855 | 866 | - | GGGTAAACAGGG |
| [**MA0157.1**](https://jaspar.elixir.no/matrix/MA0157.1) | MA0157.1.FOXO3 | 10.205409 | 0.9349451425877817 | Hk2 | 879 | 886 | - | GGAAAACA |
| [**MA0157.4**](https://jaspar.elixir.no/matrix/MA0157.4) | MA0157.4.Foxo3 | 9.145695 | 0.9057641232737347 | Hk2 | 879 | 885 | - | GAAAACA |
| [**MA0157.4**](https://jaspar.elixir.no/matrix/MA0157.4) | MA0157.4.Foxo3 | 9.145695 | 0.9057641232737347 | Hk2 | 1046 | 1052 | - | GAAAACA |
| [**MA0157.3**](https://jaspar.elixir.no/matrix/MA0157.3) | MA0157.3.Foxo3 | 9.890399 | 0.8910690505504816 | Hk2 | 876 | 887 | - | TGGAAAACAACA |
| [**MA0157.3**](https://jaspar.elixir.no/matrix/MA0157.3) | MA0157.3.Foxo3 | 9.228698 | 0.8759232598908813 | Hk2 | 1043 | 1054 | - | CAGAAAACACGA |
| [**MA0157.1**](https://jaspar.elixir.no/matrix/MA0157.1) | MA0157.1.FOXO3 | 8.3027935 | 0.871032538163635 | Hk2 | 909 | 916 | + | TGTAAAAA |
| [**MA0157.1**](https://jaspar.elixir.no/matrix/MA0157.1) | MA0157.1.FOXO3 | 8.3027935 | 0.871032538163635 | Hk2 | 920 | 927 | - | TGTAGACA |
| [**MA0157.4**](https://jaspar.elixir.no/matrix/MA0157.4) | MA0157.4.Foxo3 | 7.421585 | 0.8619980286357212 | Hk2 | 512 | 518 | + | GTAACCA |
| [**MA0157.4**](https://jaspar.elixir.no/matrix/MA0157.4) | MA0157.4.Foxo3 | 7.3680577 | 0.8606392500373811 | Hk2 | 1508 | 1514 | + | GTAAGCA |
| [**MA0157.4**](https://jaspar.elixir.no/matrix/MA0157.4) | MA0157.4.Foxo3 | 7.2678695 | 0.8580959960950424 | Hk2 | 910 | 916 | + | GTAAAAA |
| [**MA0157.4**](https://jaspar.elixir.no/matrix/MA0157.4) | MA0157.4.Foxo3 | 6.9493523 | 0.8500105159886719 | Hk2 | 920 | 926 | - | GTAGACA |
| [**MA0157.1**](https://jaspar.elixir.no/matrix/MA0157.1) | MA0157.1.FOXO3 | 7.4945745 | 0.8438828712227354 | Hk2 | 1046 | 1053 | - | AGAAAACA |
| [**MA0157.1**](https://jaspar.elixir.no/matrix/MA0157.1) | MA0157.1.FOXO3 | 7.46993 | 0.8430550181216517 | Hk2 | 922 | 929 | + | TCTACACA |
| [**MA0157.1**](https://jaspar.elixir.no/matrix/MA0157.1) | MA0157.1.FOXO3 | 7.4430075 | 0.8421506314966438 | Hk2 | 849 | 856 | - | GGAAAACT |
| [**MA0157.1**](https://jaspar.elixir.no/matrix/MA0157.1) | MA0157.1.FOXO3 | 7.4430075 | 0.8421506314966438 | Hk2 | 1785 | 1792 | + | GGAACACA |
| [**MA0157.3**](https://jaspar.elixir.no/matrix/MA0157.3) | MA0157.3.Foxo3 | 7.62586 | 0.8392356400163588 | Hk2 | 908 | 919 | + | ATGTAAAAATAA |
| [**MA0157.3**](https://jaspar.elixir.no/matrix/MA0157.3) | MA0157.3.Foxo3 | 7.5830145 | 0.8382549369520332 | Hk2 | 510 | 521 | + | CAGTAACCAGAT |
| [**MA0157.3**](https://jaspar.elixir.no/matrix/MA0157.3) | MA0157.3.Foxo3 | 7.5260916 | 0.8369520187847072 | Hk2 | 1506 | 1517 | + | GAGTAAGCAGCA |
| [**MA0157.4**](https://jaspar.elixir.no/matrix/MA0157.4) | MA0157.4.Foxo3 | 6.333969 | 0.8343891678291692 | Hk2 | 643 | 649 | - | AGAAACA |
| [**MA0157.1**](https://jaspar.elixir.no/matrix/MA0157.1) | MA0157.1.FOXO3 | 6.9990487 | 0.827237182162998 | Hk2 | 407 | 414 | + | TGGAAACT |
| [**MA0157.3**](https://jaspar.elixir.no/matrix/MA0157.3) | MA0157.3.Foxo3 | 6.469322 | 0.8127634460402934 | Hk2 | 917 | 928 | - | GTGTAGACATTA |
| [**MA0157.4**](https://jaspar.elixir.no/matrix/MA0157.4) | MA0157.4.Foxo3 | 5.3412256 | 0.809188621195553 | Hk2 | 1086 | 1092 | - | GGAAATA |
| [**MA0157.1**](https://jaspar.elixir.no/matrix/MA0157.1) | MA0157.1.FOXO3 | 6.406073 | 0.8073179630312998 | Hk2 | 1086 | 1093 | - | GGGAAATA |
| [**MA0157.1**](https://jaspar.elixir.no/matrix/MA0157.1) | MA0157.1.FOXO3 | 6.3661747 | 0.8059776973244528 | Hk2 | 715 | 722 | + | GGAAAAGA |

所有结合位点均做缺失突变

>Hk2-promoter(Cavia,mt) 1873bp

AGCCTCTGGAGTGCTGGGATTACAGGCGTGCACCACCACGGCAGGCTCAGTTTGTATTTGGAGACTACAGGGCTCAGGGTGGAGGTGGGCAAGACCTGAAGCAGGATGAGGGGGCATTGACTCTCCTGTGCTCCTTGGTGATATGGATAGGAGGTGTCAGTGCGGAGAGAGGTGGTCAGCCCCTGGATCTCCAACAGGCCATACAGACTGCTTGCTGCCCACTGATGGGTGTGAAGGTGACAGGCAGCAAGGAATCAAGGATGGCTTGAGTTGGAGGGAAATGTCATTTTCTGAGACAACTGGAACAGCAAAAGATTTGGGAAGAAAAATCTACTTCACCTTTGCATTAAGTGTGACATGCTAGTCACTTCAGTGAAGATGACAAATGTCAGTTGGAGGGATAGGCCTGGGAGAGGTTAGTTGTAGATTTACTTTTGTGAGTCACCCATGTGTTGGAGATATTACCAGTCATAGGGCTGGGAAGGATCATCCAGGGAGAGCCAGGGAGTAGGGTGGAAGGCAGCTTTGGAATTCTACAGCACTTTTCAGCAGAGACCACGGGGCTGAGGAGCTTGGGGGAGTGTGGCAGGACTGGAACCCAGCAGAGCAAGAGCAAGGAAGAGTGTCTGGATGGAGGATGCGGTCTCGTCCCCACTGTCACCGAGAGATGGAATAATGCCAGGATGGCAGCATAAGATTTGCAGCGTGTAGGTCATTGCAATTAGGAACAAAGTACTTTGGACCCCATCCTGACAGGCAAGGGACGGAAGGATGGGTATTTGGTAAGTTTTGCTGAGAGGCTGAATCATGGATATATAGTATACAAAAAGCATTTATTTCTCTATTGCAGAGCACTCCACTGAGAAAAACTCTTCTGTAGAACACGGCAGACCTTTCCTTCCTTCCACTGTGACTACTCCAATCCCAGGCTTATCTTTCTCCACTAGTATCAATCAGGTTTGTATTTCGTTGCCACTCCACCAACATTTGGAGTTGGGTGCCAGGCACTAGGTCTGGCTGGGGCTCCTGGTCTTCGAAGCATCTGTAAGCCACCCCGCCCAAACGAGTCCCACACATGCCCAGATCATCCAGTTACACACATACCATACACCTAGTTTCACCCACTTATCAGCCCCACAGCTGCAGACAACGTCTCTTTCCCACCATCTACTTCCCTGGCTTCTAGAAAGACGTGATACTATAAAGGGGAAGCGATAGAGCCTTACATCAGAGGCATATGGGTCTTTTTCTCCATCTGCAACTTGCTGGCTGTGTGATGTTGGGAAAATTTAACATTGCTGTGCCTCGATTCTGTATTTGTAAAGCGGGGTTGATGAAACTCACATTCCTATCAGCGATGTGAGGGGTAGATGGCCAGACCTTTATAATCTCGGTGTAGCTAAGGCGGCTCACTTTGCATCAGCGATGCGTGGACAGGTCGAGCCAGAATAGGCTACAACAAGGATGCTTCAGATGTGTGATGGAGCCCCTCATGTGATCCCGCGACACCAGACGGTGTATCTCTGCACAGGGTGTGTGCTAGTCCAATGACTTGTCTTTCCAGTAGTCCCACCTCTCCTGGGTCGGCGATCACGCGCCCCCCACCCGTAGCTGGGGCTGACGCGGCTGTGGCTCATGCGCCTTTGCGTCCCAGCTTCATGCCACCGTCCCATCCTGAGCGCCAGACCCCGCCCAGCGCCCCAGGCGCGCCTCTCAAGCCAGCCGGCTCGGGTGTCGGAGTAGCCGCGCCCGCGGGTCGTGCGCGCTGATTGGCTGTGCCGCGCCGATGGGCGGCCCGCGGGCGCACACACCCTCTCTGTGCAGCCAATAGGAGAGCGCACGTCACTGAGCGGGCGGCCCGCGAGCCGGCAGC
